# Establishment of Repertoire of Placentome-Associated MicroRNAs and Their Appearance in Blood Plasma Could Identify Early Establishment of Pregnancy in Buffalo (*Bubalus bubalis*)

**DOI:** 10.3389/fcell.2021.673765

**Published:** 2021-08-26

**Authors:** Parul Sarwalia, Mustafa Raza, Apoorva Soni, Pratiksha Dubey, Rajeev Chandel, Rakesh Kumar, A. Kumaresan, Suneel Kumar Onteru, Ankit Pal, Kalpana Singh, Mir Asif Iquebal, Sarika Jaiswal, Dinesh Kumar, T. K. Datta

**Affiliations:** ^1^Animal Genomics Laboratory, Animal Biotechnology Centre, National Dairy Research Institute, Karnal, India; ^2^Centre for Agricultural Bioinformatics, Indian Agricultural Statistics Research Institute, New Delhi, India; ^3^Biological Science Laboratory, Indian Institute of Science Education and Research, Mohali, India; ^4^Animal Biochemistry Division, National Dairy Research Institute, Karnal, India; ^5^Theriogenology Laboratory, SRS of National Dairy Research Institute, Bengaluru, India

**Keywords:** circulatory miRNA expression, buffalo placenta, early pregnancy detection, feto-maternal cross talk, maternal recognition of pregnancy

## Abstract

Precise early pregnancy diagnosis in dairy animals is of utmost importance for an efficient dairy production system. Not detecting a dairy animal pregnant sufficiently early after the breeding results to extending the unproductive time of their milk production cycle and causes substantial economic loss for a dairy producer. At present, the most conventional and authentic pregnancy confirmation practice in cows and buffaloes is rectal palpation of the reproductive organs at Days 35–40 after insemination, which sometime leads to considering an animal as false pregnant. Other alternative methods available for early pregnancy diagnosis lack either accuracy or reproducibility or require elaborate instrumentation and laboratory setup not feasible to practice at farmers’ doorstep. The present study was aimed at establishment of the microRNA (miRNA) repertoire of the placentome in buffaloes, which could capture the event of the cross talk between a growing embryo and a dam, through fetal cotyledons and maternal caruncles, and thus could hint at the early pregnancy establishment event in ruminants. Total RNA was isolated from buffalo placentome tissues during early stages of pregnancy (at Day < 25 and Days 30–35), and global small RNA analysis was performed by using Illumina single-end read chemistry and *Bubalus bubalis* genome. A total of 2,199 miRNAs comprising 1,620 conserved and 579 non-conserved miRNAs were identified. Stringent functional miRNA selection criteria could predict 20 miRNAs worth evaluating for their abundance in the plasma of pregnant, non-pregnant, cyclic non-bred, and non-cyclic prepubertal animals. Eight of them (viz., miR-195-5p, miR-708-3p, miR-379-5p, miR-XX1, miR-XX2, miR-130a-3p, miR-200a-3p, and miR-27) displayed typical abundance patterns in the plasma samples of the animals on Day 19 as well as Day 25 post-insemination, thus making them ambiguous candidates for early pregnancy detection. Similarly, higher abundance of miR-200a-3p and miR130a-3p in non-pregnant animals was indicative of their utility for detecting the animals as not pregnant. Most interestingly, miR-XX1 and miR-XX2 were very characteristically abundant only in pregnant animals. *In silico* target prediction analysis confirmed that these two miRNAs are important regulators of cyclooxygenase-2 (COX-2) and cell adhesion molecule-2 (CADM-2), both of which play a significant role in the implantation process during feto-maternal cross talk. We interpret that circulatory miR-XX1 and miR-XX2 in blood plasma could be the potential biomarkers for early pregnancy detection in buffaloes.

## Introduction

Profitable milk production depends on regular calving of dairy animals, with an ideal target of one calf born every year from one animal. To attain this target in a dairy farm, the imperative prerequisites are efficient reproductive management and accurate assessment of each reproductive event of dairy animals, such as estrus, pregnancy, parturition, and postpartum events. Among premier dairy animals, buffaloes play a major role in developing countries. For instance, India ranks highest in the world for its milk production with a total of 302.3 million bovine population, out of which 192.5 million are cattle and 109.9 million are buffaloes (Livestock Censuses, Department of Animal Husbandry, Gol), which contribute to more than 50% of the milk production in India (Basic Animal Husbandry Statistics, DAHD&F, Gol). However, the reproductive potential of buffaloes is limited by certain problems, like late puberty, poor estrus expression in summer seasons, extended calving interval, and high incidence of anestrous (Zicarelli, 2010; de Carvalho et al., 2016). These problems, especially the anestrous and repeat breeding, lead to an estimated annual loss of 20–30 million tonnes of milk production, which corresponds to a loss of almost $6,836.44 million. Similarly, lack of an early pregnancy detection method also causes economic loss to buffalo dairy farms. In the field practice, a farmer depends on artificial insemination (AI) for breeding an animal, but at the same time, it is notable that a single insemination would not necessarily mark the animal as pregnant until a field-applicable early pregnancy detection method is available. Generally, farmers wait for more than 35 days for pregnancy diagnosis in dairy animals. It is well-known that the most conventional and authentic practice for pregnancy confirmation in cows and buffaloes is rectal palpation, where the detection can be done at the earliest by 35–40 days after breeding (Romano et al., 2007). However, per-rectal examination sometimes leads to early embryonic mortality in buffaloes. Hence, an early pregnancy detection method close to 21 days post-insemination is essential, so that the farmer would not miss the consecutive estrus event. Missing even a single heat/estrus delays the calving by 21 days, thereby resulting to an economic loss of almost $86.14 per animal, corresponding to the cost of 21 days of milk production. Although some indirect pregnancy detection methods based on the measurement of the progesterone, estrone sulfate, interferon-tau, and pregnancy-associated glycoproteins were reported (Balhara et al., 2013), none of these methods qualify as an ideal early pregnancy diagnosis method due to various limitations, such as lack of accuracy, poor reproducibility, requirement of elaborate instrumentation, and laboratory setup. Therefore, an accurate early pregnancy detection method needs to be developed for buffaloes.

During early stages of the pregnancy establishment in mammals, the growing embryo and the maternal counterpart, such as uterine endometrium, establish a cross talk with each other through extensive structural and functional changes, which result in the formation of a vital pregnancy-associated organ, known as placenta. The placenta undergoes rigorous growth and development during the whole gestation period. It facilitates the cross talk between mother and fetus and thus helps in nutrient and gaseous exchange between the two during the pregnancy. Different mammals exhibit different types of placenta. Among them, cotyledonary placenta is common in ruminants, including cattle and buffalo. Numerous molecular interactions involving many biomolecules play several roles during the formation of this kind of placenta between maternal caruncles and fetal cotyledons. The bidirectional signaling messages between the fetus and mother through a wide range of macromolecules play a vital role in the establishment of pregnancy. One such class of biomolecules is microRNAs (miRNAs), which not only is involved in the placenta formation but also promotes embryo development.

MiRNAs are 18- to 22-nucleotide short non-coding RNAs regulating the gene expression either by mRNA cleavage, de-adenylation, and inhibition of translational repression or by targeting the gene promoters. Primarily, miRNAs regulate the biological processes (BPs), such as cellular differentiation, proliferation, and apoptosis (Wang et al., 2012). Intensive studies in human (McCallie et al., 2010; Xu et al., 2011; Rosenbluth et al., 2013), bovine (Tesfaye et al., 2009), and murine (Tang et al., 2007; Yang et al., 2008) gametes and embryos predicted their role in various pathways involved in early embryonic development. In addition, miRNAs are the newly identified key players among the molecules interplaying to achieve a successful implantation process (Paul et al., 2018; Balaguer et al., 2019; Kurian and Modi, 2019; Reza et al., 2019). Hence, the miRNA might play a pivotal role in establishing communication between embryo and the uterus (Gross et al., 2017; Battaglia et al., 2019). Furthermore, the miRNAs were also found in the uterine luminal fluid and in embryo culture media. They can be easily entered into peripheral circulation of dam either in free form or bound form or in the packaged nano-structures called exosomes. Hence, the miRNA from the placentome can be recognized in maternal circulation. Therefore, detection of placentome-related miRNA in maternal blood could be one of the possibilities to identify early pregnancy. Supporting this hypothesis, a few studies quantified some pregnancy- and estrous-related circulatory miRNAs in humans, cattle buffalo, pigs, and ewes, etc. (Yong and Chan, 2020) in the plasma. However, none of these studies were able to explain the site of origin of these miRNAs and their significance in regulating feto-maternal cross talk. The present work aimed to identify and establish the miRNA repertoire associated with buffalo placentome during early pregnancy event and to identify them in maternal plasma for accurate detection of early pregnancy in buffaloes.

## Materials and Methods

### Tissue Sample Collection

**FIGURE 1:**
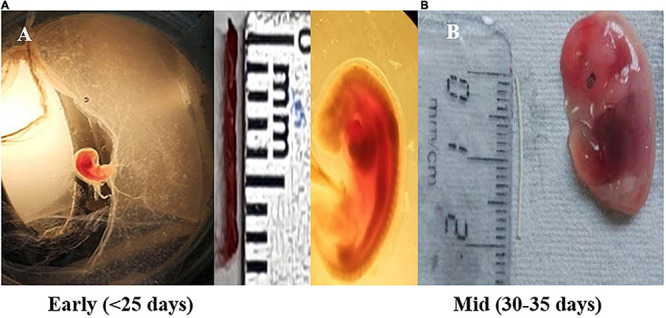
Fetal size depicting early pregnancy and mid-pregnancy (shown in scale, cm). **(A)** Fetus measure between 0.8 and 1.1 cm in length (crown rump length) corresponds to Day < 25 of gestation (early stage) and shows the formation of the heart and primitive intestine. **(B)** Embryo having length of 1.6–2.0 cm (crown rump length) corresponds to Day ±31–35 days of gestation (mid-stage). During this, the development of external ear takes place along with the tail representing, one-fourth of the total length of the embryo.

Gravid uterus of female pregnant buffalo (*Bubalus bubalis*) (*n* = 4) from a slaughterhouse were included in the experimental study. All the animals considered here underwent a thorough antemortem examination by a veterinarian, and apparently, only healthy animals were slaughtered. The uterine horn was incised longitudinally, and buffalo placental tissues–caruncle [maternal placentome (MP)] and small button-like tissues depicting future cotyledons [fetal placentome (FP)]–were collected from the pregnant horns of the animal. For placental tissues, the samples were paired samples from the maternal and fetal parts of the uterine placentome of the same animal. Size of the crown rump length (CRL) of the fetus was used as a reference to calculate the stage of pregnancy (Figure 1; Edward, 1965; Soliman, 1975; Morini et al., 2016). Based on CRL, tissues from the animals of Day 25 and Days 30–35 of pregnancy were collected in cold Dulbecco’s phosphate-buffered saline (DPBS) and washed and stored in RNA later (Fajardy et al., 2009; Aliniyaa et al., 2017).

### RNA Isolation From the Collected Tissues

Tissue samples were homogenized with TOMY Micro smasher_MS100 (Tomy Seiko Co., Ltd., Tokyo, Japan) and lysed with 300 μl of lysis/binding buffer. Homogenate additive was added to the lysate at one-tenth of the lysate volume followed by addition of acid phenol–chloroform. After brief vortex and centrifugation, the upper aqueous phase was aspirated into a new vial. This aqueous phase was mixed with 1.25 volumes of 100% ethanol and loaded onto a filter cartridge. The remaining steps of the purification were followed as per manufacturer’s guidelines including on-column DNase treatment (Qiagen Inc., Valencia, CA, United States; Cat#79254) (Horvath et al., 2007; Mormann et al., 2010). RNA was eluted in 25 μl of nuclease-free water (Ambion, Austin, TX, United States; Cat#AM9938). Eluted RNA was stored at −80°C until further use.

The concentration and purity of the extracted RNA were evaluated using the Nanodrop Spectrophotometer (Thermo Fisher Scientific, Waltham, MA, United States; 2000). The integrity of RNA was analyzed on the bioanalyzer (Agilent Technologies, Santa Clara, CA, United States; 2100 expert). A total of four RNA samples from early (25 days) and mid (30–35 days) stages of pregnancy for both MP and FP showing >6 RNA integrity number (RIN) value were processed for small RNA sequencing (SmRNA).

### Library Preparation and Small RNA Sequencing

Small RNA sequencing library was prepared with TruSeq Small RNA Sample Preparation protocol (Illumina, San Diego, CA, United States) at Genotypic Technology Pvt. Ltd. (Bangalore, India). Total RNA of 1,000 ng was used as starting material. The 3′ adapters were ligated to the specific 3′OH group of enriched miRNAs followed by ligation of 5′ adapter. Adapter ligated fragments were reverse transcribed with Superscript III reverse transcriptase (Invitrogen, Carlsbad, CA, United States) (Krug and Berger, 1987) by priming with reverse transcription primers. cDNA thus formed was enriched and barcoded by PCR amplification (15 cycles). Amplified library was size selected using polyacrylamide gel.

Illumina Universal Adapter used in the study was 5′-AATGATACGGCGACCACCGAGATCTACACGTTCAGAGTT CTACAGTCCGA-3′ and Index Adapter was 5′-CAAGCAGAA GACGGCATACGAGAT [INDEX] GTGACTGGAGTTCCTTG GCACCCGAGAATTCCA-3′. [INDEX]–Unique sequence to identify sample-specific sequencing data.

Library was size selected in the range of 140–160 bp followed by overnight gel elution and precipitation in the presence of glycogen, 3M sodium acetate (Sigma, St. Louis, MO, United States), and absolute ethanol. Pellet was resuspended in nuclease-free water (Invitrogen, Whitefield, Bangalore, India). Illumina-compatible sequencing library was quantified by Qubit fluorometer (Thermo Fisher Scientific, Waltham, MA, United States), and its fragment size distribution was analyzed on Agilent 2100 bioanalyzer.

Illumina single-end replicated reads were generated for *B. bubalis* samples (Supplementary Table 1). In the record, FP and MP correspond to FP (cotyledon) and MP (caruncle) of buffalo. These replicated raw data of small RNA (sRNA) were in FASTQ format and used further for buffalo miRNA analysis.

### Preprocessing of Data

Raw sRNA data quality was visualized through FastQC program v0.11.5^1^ (Andrews, 2010) showing adapters and some reads having low Phred quality score. The 3′ Illumina adapters were removed using Fastx tool v0.0.13^2^ (Hannon, 2010). After adapter removal, sRNA reads were subjected to quality filter set with Phred score of ≥20. The FastQ reads were converted into FASTA by Perl script, and reads were further filtered considering only those sequences that are having sequence length of 16–30 bp. The reads having quality and length less than the above-mentioned parameters and reads that include tRNA/rRNA were discarded. High-quality reads were selected for further downstream analysis. Finally, filtered reads were aligned by utilizing bowtie2 (Langmead and Salzberg, 2012) with buffalo genome UOA_WB_1 Assembly 1.0 (Low et al., 2019). After alignment, unaligned reads were discarded, and aligned reads were used for further analysis.

### Identification of MicroRNA

Identification of miRNA was carried out by using the UEA sRNA Workbench miRCat tool with default parameters (Stocks et al., 2018). The miRCat tool (Paicu et al., 2017) identified miRNAs by using the reference genome of buffalo.^3^ The identified miRNAs having the abundance > 5 were selected. Conserved (known) and non-conserved (novel) miRNAs were identified by using UEA sRNA Workbench miRProf tool. miRProf requires a set of sRNA samples and a genome file for input to execute against the mature miRNA sequences from miRbase (Griffiths-Jones et al., 2006).^4^

### Differential Gene Expression Analysis

Differential expression analysis of miRNA was done through DESeq2 tool (Love et al., 2014). DESeq2 Bioconductor package was used with filtering criteria of *p*-value < 0.05 and log fold change2 of gene expression levels to retrieve significant differentially expressed miRNAs. This tool implements a range of statistical methods based on the negative binomial distributions. MiRNAs having fold change value of 2 and above were considered as upregulated, whereas those having fold change below −2 were considered as downregulated. MiRNAs with fold change values greater than −2 and less than 2 were considered as normally expressed miRNAs (Wang et al., 2019).

### Comparative Analysis of Water Buffalo MicroRNAs With Other Ruminants

Comparative analysis of water buffalo miRNAs with other ruminants in RumimiR database having 19,846 miRNAs of three species was performed. The miRNAs were extracted and matched with all miRNAs downloaded from RumimiR database (Bourdon et al., 2019) using the in-house Perl script to find conserved and water buffalo-specific novel miRNAs. The unmatched miRNAs were considered to be novel miRNAs extracted within the study.

### Potential Messenger RNA Target Identification

A set of the sRNA samples along with the genome file were used as an input file in miRanda tool for the identification of miRNA targets. miRanda focuses on sequence matching of miRNA:mRNA pairs by calculating energy of physical interaction, and therefore, the significant miRNA was selected on the basis of its score and free energy (Miranda et al., 2006). Furthermore, PANTHER classification system was used for top-ranked predicted genes to identify associated BPs.

### Blood Sample Collection

Buffaloes falling under different experimental groups, viz., pregnant, cyclic non-pregnant, cyclic heifer, and prepubertal animals (*n* = 5 each), were included for blood sample collection. In the first group (pregnant), blood samples were collected on Day 7 (counted from the day of insemination), Day 11, Day 15, Day 19, Day 25, and Day 30. Confirmation of pregnancy was done using the rectal palpation on Day 45 of AI. In the second group, cyclic buffaloes were not inseminated at estrus, and blood samples were collected from these animals on Day 7, Day 11, Day 15, Day 19, Day 25, and Day 30 post-estrus to maintain uniformity with the blood sampling timings in pregnant animals. These animals showed estrus after 20–22 days of previous estrus but were not taken into account, and blood collection was continued till Day 30 of previous estrus. In this case, Days 25 and 30 could be Day 5 ± 3 and Day 10 ± 3 days of the second estrous cycle. However, the animals were not pregnant, as they were not bred at both the estruses. Thus, they also represent the non-pregnant samples.

In the third group, cyclic heifers were used in order to rule out the effects of previous pregnancies, which might be possible in group 2, as they were adult buffaloes that calved earlier but used in the experiment after voluntary waiting period or during breeding period on miRNA. Again, they were not bred at both the estruses. Blood samples were collected as mentioned earlier for the second group.

In the fourth group, prepubertal animals were used as a true negative control, as they have not started cyclicity, never exposed to semen, or have never been pregnant. This group was considered important to ascertain the specificity of the miRNA to pregnancy.

Briefly, around 10 ml of blood was collected in BD-K2 EDTA vacutainer with the help of a 21-gauge needle. Samples were allowed to settle down for 15 min at room temperature, then put in ice in a cooler, and transported to the laboratory within 1 h after collection. Tubes were centrifuged (1,000×*g* for 10 min) in a refrigerated centrifuge, and serum was distributed into 0.5-ml aliquots and stored at −80°C until further processing.

### Small RNA Isolation and cDNA Preparation

Small RNA including the miRNA was purified from the plasma samples using the miRNeasy serum/plasma advanced kit from Qiagen (Hilden, Germany) (Cat#217204) according to the manufacturer’s protocol (Kloten et al., 2019; Wright et al., 2020). Total RNA for each sample was reverse transcribed using miScript II RT kit Qiagen according to manufacturer’s protocol. A HiSpec buffer (5×) was used to prepare cDNA for mature miRNA profiling. Subsequently, the cDNA was diluted in nuclease-free water (5 ng/μl for q-PCR) and stored at −20°C.

### Buffalo Mature MicroRNA Expression Profiling

With the use of the quantitative real-time PCR method, the relative miRNA abundance of the 20 most differentially expressed miRNA obtained from the next-generation sequencing (NGS) data was identified in the prepared cDNA. The specific primer for the conserved miRNA was designed automatically using miRPrimer tool, using the most current miRNA genome, as annotated in miRBase V20.^5^ For the non-conserved miRNA, the primers were designed manually by extracting their mature seed sequence from the NGS-based sequencing results, then replacing the uracil in the sequence with thymine, and further adjusting the guanine–cytosine (GC) content and their melting temperature by adding the suitable combination of cytosine, adenosine, and guanine nucleotide. Controls included were btamiR-423 and btamiR-103 (Supplementary Table 3).

For real-time PCR, miScript SYBR Green PCR kit containing miScript Universal Primer as reverse primer and QuantiTect SYBR Green PCR Master Mix (Qiagen) were used, and reactions were designed as per manufacturer’s protocol. Amplification was programmed in a CFX-96 instrument (Bio-Rad Laboratories, Hercules, CA, United States). Cycling conditions used for real-time PCR included an initial activation step at 95°C for 15 min to activate HotStar Taq DNA polymerase and 40 cycles of denaturation (at 94°C for 15 s), annealing (at 55°C for 30 s), and extension (at 70°C for 30 s). Fluorescence data were collected at the holding stage of the extension step. Specificity and identity were verified by melting curve analyses. Threshold cycles values (CT) were obtained and were assessed using GraphPad Prism8 for statistical analyses.

### Relative MicroRNA Expression Analysis

Raw CT values of the samples were extracted in Excel version [1997–2003 (.XLS file format)]. Geometric mean of the CT values of btamiR-423-5p and btamiR-103 was used to calibrate the CT values of the samples at different days of pregnancy. Global CT mean of Day 7 for a particular miRNA was used to normalize the CT values obtained for that specific miRNA at subsequent days of pregnancy. The distribution of CT values and raw data average for a particular miRNA in all the days of pregnancy were reviewed. Average ΔCT, 2^–ΔCT^, and fold regulation were calculated based on Schmittgen and Livak (2008). Average CT values converted in 2^–ΔCT^ values were used in graphical representation; and *p*-values (statistical significance was set at *p* < 0.05) were estimated using GraphPad Prism8 using two-way and one-way ANOVAs with Tukey’s *post hoc* correction. To evaluate the diagnostic potential value of circulating miRNAs in predicting their diagnostic potential, receiver operating characteristic (ROC) curves were constructed and area under the curve (AUC) was calculated in the plasma samples of pregnant and non-pregnant animals by GraphPad Prism software Version 5.

## Results

### Small RNA Sequencing of Maternal and Fetal Placentome at Different Days of Pregnancy

**FIGURE 2:**
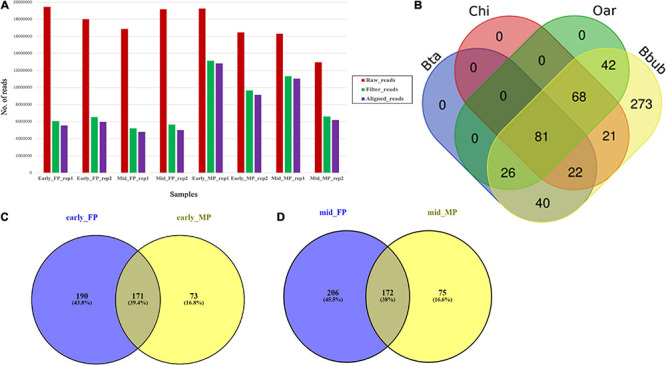
**(A)** Abundance of raw, trimmed, and aligned reads in early and mid caruncular (MP) and cotyledonary (FP) placentome samples. **(B)** Distribution of conserved miRNAs in different ruminant species (Bta, *Bos taurus*/cattle; Chi, *Capra hircus*/goat; Oar, *Ovis aries*/sheep, Bbub/buffalo) using RumimiR database. **(C)** Venn diagram for miRNAs identified in early-stage fetal placentome early_FP (cotyledon) and maternal placentome early_MP (caruncle). **(D)** Venn diagram for miRNAs identified in mid-stage fetal placentome mid_FP (cotyledon) and maternal placentome mid_MP (caruncle).

Global SmRNA produced the raw reads ranging from 12.6 to 19.4 million in the fetal cotyledons and maternal caruncles (Figure 2A). More than 90% of the total raw reads from the fetal cotyledon (91.34% in FP 1 (FP1) and 90.36% in FP2) were aligned with the *B. bubalis* genome. Similarly, more than 95% of the 96.08 and 95.79% of the total reads from the maternal caruncle or MP, i.e., 96.08% from MP1 and 95.79% from MP2, were aligned with the buffalo genome. FP1 and MP1 refer to the early stage of pregnancy, and FP2 and MP2 refer to the mid-stage of pregnancy (Supplementary Table 1). Among the aligned reads, only less than 4% of the total reads belong to other ncRNAs, like rRNAs, tRNAs, snoRNAs, and snRNAs.

### Identification of the Conserved/Known and Non-conserved/Novel MicroRNAs in Maternal and Fetal Placentome at Different Days of Pregnancy

Analysis of all the aligned reads from FP1, FP2, MP1, and MP2 samples (two replicates at each stage) revealed a total of 2,199 miRNAs, out of which 1,620 were known and 665 were novel. The secondary structure prediction analysis on 665 novel identified miRNAs found that 86 miRNAs had the star (^∗^) sequence, indicating that these miRNAs originated from the same hairpin structure of the main miRNAs including the pri-/pre-miRNA (Supplementary Table 2). On an average, the FP samples have a significantly (*p* < 0.01) higher number of miRNAs (315.75 ± 19.81) than the MP samples (234 ± 5). Accordingly, the novel miRNAs and the miRNAs with star sequence were higher in the FP than the MP.

#### Comparative Analysis of Water Buffalo MicroRNAs With Other Ruminants

Among the total 2,199 miRNAs, 573 miRNAs were selected for comparative analysis after removing redundancy. Comparative analysis with 19,846 miRNAs available at RumiRBase revealed that water buffalo shared 169, 192, and 217 miRNAs with the bovine, caprine, and ovine, respectively. Interestingly, our study revealed a new piece of information that 273 miRNAs are novel miRNAs specific to water buffalo. Distribution of the shared and unique miRNAs among four ruminant species is shown in Figure 2B.

#### Conserved/Known and Non-conserved/Novel MicroRNA Associated With Caruncle (Maternal Placentome)

A total of 293 miRNAs were identified in MP1 and MP2. Among them, 270 were conserved/known and 23 were non-conserved/novel. Here, 46 miRNAs were specific to early-staged MP (MP1), out of which 38 were conserved/known and eight were non-conserved/novel. Similarly, 49 miRNAs were specific to the mid-staged MP (MP2), out of which 39 were known and 10 were of novel category. Also, from the list of 198 miRNAs that were commonly expressed between the two stages, 193 were conserved/known and five were non-conserved/novel (Supplementary Figure 1).

#### Conserved/Known and Non-conserved/Novel MicroRNA Associated With Cotyledon (Fetal Placentome)

A total of 487 miRNAs were identified in FP1 and FP2 samples. Among them, 231 were known miRNAs and 256 were novel miRNAs. Furthermore, to ascertain how many of these belong to early stage and mid-stage of pregnancy, we found that 109 miRNAs were exclusively associated with early-staged FP, out of which 82 were known and 27 were novel. The identified 109 miRNAs were of great interest because they are the early signatures from the fetal side to initiate fetal–maternal communication and, hence, begin the cross talk to further promote the growth of the embryo. Likewise, among 126 miRNAs associated with mid-staged FP, 18 were conserved/known and 108 were non-conserved/novel. Further dwelling into the data, we observed that nearly 252 miRNAs were common in both early and mid FP samples, out of which 186 were known and 66 were novel (Supplementary Figure 2).

#### MicroRNA Common Between Cotyledon (Fetal Placentome) and Caruncle (Maternal Placentome)

The comparison of early FP and early MP (FP1 and MP1) revealed that 171 miRNAs (145 were known and 26 were novel) were common in both, 190 were specific to FP1, and 73 were specific to MP1 (Figure 2C). More interestingly, out of the 171 common miRNAs in early FP and early MP, 70 miRNAs (66 known and four novel miRNAs) had the mature star (^∗^) sequence. Similarly, in case of mid-stage of pregnancy, 172 miRNAs (145 were known and 27 were novel) were commonly expressed in both the FP and MP (Figure 2D).

### Significantly Differentially Expressed MicroRNA Between Cotyledon and Caruncle at Different Stages of Pregnancy

**FIGURE 3:**
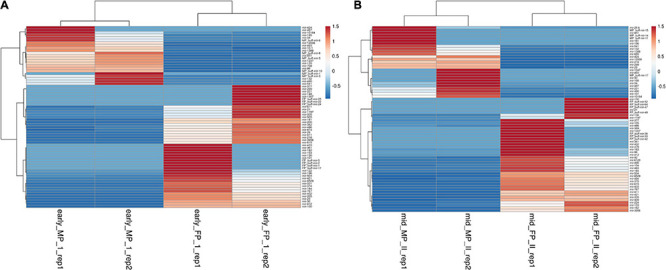
Heat map for the differentially expressed miRNAs between fetal and maternal placentome during. **(A)** Early stage of pregnancy and **(B)** mid-stage of pregnancy, where red color depicts upregulated miRNA and blue color depicts miRNA that are downregulated. The scale bar from −0.5 to 1.5 corresponds to the fold change.

Using the DESeq algorithm, we generated the heat map of differentially expressed miRNAs at the comparison between the stages, namely, FP1 and MP1, FP2 and MP2 (Figures 3A,B), MP1, MP2, and FP1 and FP2 (including the replicates; Supplementary Figures 3, 4). These comparisons were designed to shortlist those miRNAs that depicted the fold difference between FP and MP at different stages of pregnancy. A total of 71 and 70 miRNAs were significantly differentially expressed (SDE) with log ≥ 2 fold change between FP1 and MP1 and between FP2 and MP2, respectively. Among these SDE miRNAs, four upregulated (miR-148a, miR-30a-5p, miR-195-5p, and miR-XX2) and four downregulated (miR-708-3p, miR-127, miR-200a-3p, and bubmiR-55) miRNAs in MP were selected. Similarly, six upregulated (miR-379-5p, miR-130a-3p, miR-1307-3p, miR-369-5p, miR-143, and miR-27) and six downregulated (miR-181b-5p, miR-23b-3p, miR-487a-3p, miR-XX1, bubmiR-1, and miR-660) miRNAs associated with fetal cotyledon were considered for further validation in the plasma samples of pregnant, non-pregnant, cyclic heifer, and prepubertal animals (Supplementary Tables 4, 5). Taken together, the list of SDE provided 20 candidate miRNAs for further miRNA abundance analysis in the plasma samples.

Furthermore, for novel miRNA where bubmiR-1 and bub-miR-55 were shortlisted from the list, their secondary structures were also predicted using the web-based interface called RNAFold-6. The hairpin structure basically depicts the mature seed sequence that is responsible for generating the regulatory role (Supplementary Figures 5, 6).

### MicroRNA Abundance Validation in Plasma Through RT-qPCR

**FIGURE 4:**
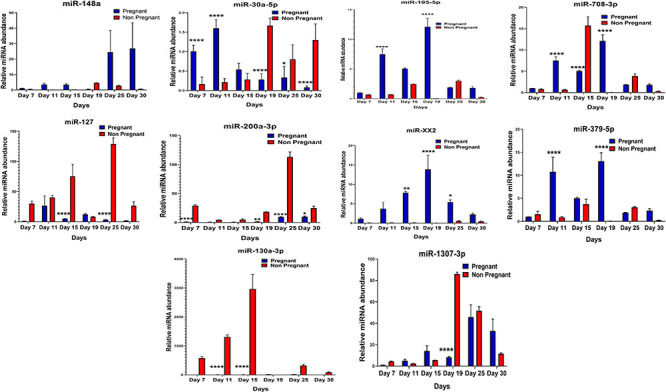
Graphical representation of the abundance of shortlisted miRNAs in the blood plasma of pregnant and non-pregnant animals. Here, X and Y axis represents the relative miRNA abundance and days. Blue and red columns represent pregnant and non-pregnant animals. *p < 0.05; **p < 0.01; and ****p < 0.0001.

**FIGURE 5:**
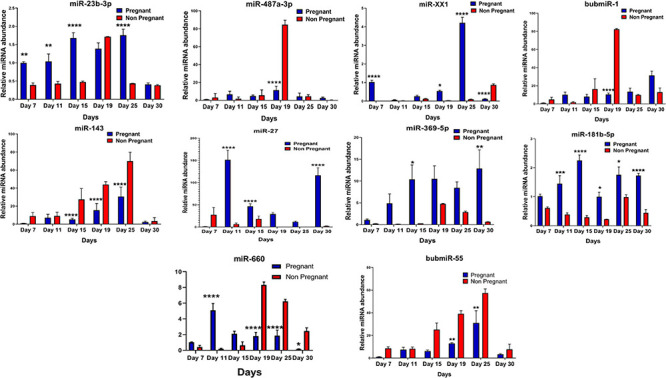
Graphical representation of the abundance of shortlisted miRNAs in the blood plasma of pregnant and non-pregnant animals. Here, X and the Y axis represents the relative miRNA abundance and days. Blue and red column represents pregnant and non-pregnant animals. *p < 0.05; **p < 0.01; ***p < 0.001; and ****p < 0.0001.

It was observed that out of 20 shortlisted miRNAs, miR-195-5p, miR-XX2, miR-708-3p, miR-379-5p, miR-27, miR-130a-3p, miR-XX1, and miR-200a-3p were significantly different in the plasma samples of pregnant versus non-pregnant animals at Day 19 and Day 25 of pregnancy. The period of Day 19 to Day 25 corresponds closest to the window of implantation in ruminants. However, in order to evaluate their dynamic presence during the course of pregnancy, we also assessed the candidate miRNA in circulation on Day 7, Day 11, Day 15, and Day 30 of pregnancy. Interestingly, among the known miRNAs, miR-1307-3p, miR-487a-3p, miR-30a-5p, miR-660, miR-143, miR-200a-3p, and miR-130a-3p were significantly higher in the non-pregnant group at Day 19 and Day 25 of pregnancy. Although this increase in their expression within the non-pregnant group minimized their chances of qualifying in the category of pregnancy-associated signature, at the same time, it built a strong inference that their higher abundance in circulation might be the leading cause for pregnancy failure in animals (Figures 4, 5).

Furthermore, the expression of miR-27, miR-XX1, miR-379-5p, miR-369-5p, miR-181b-5p, miR-23b-3p, and miR-148a is higher in the pregnant group as compared with the non-pregnant group (Figures 4, 5). Though these selected miRNAs have significantly higher expression in the pregnant group at Day 19 and Day 25 of pregnancy, it is of utmost importance to see if they show the same abundance in the experimental control groups as well. While keenly observing the data for subsequent days of pregnancy, we also observed a particular trend for these miRNAs. In order to understand the dynamicity in their expression, the graphs of these miRNAs were further classified in four different categories, which are as follows:

**Category A:** MiRNA having high expression in the pregnant group at Day 19 but reversing the trend by having higher expression in the non-pregnant group at Day 25.
**Category B:** MiRNA having high expression in the pregnant group at both Day 19 and Day 25 of pregnancy and very low abundance in the non-pregnant group at both days.
**Category C:** MiRNA having high expression in non-pregnant group.
**Category D:** MiRNA having high expression in pregnant and prepubertal groups.

**FIGURE 6:**
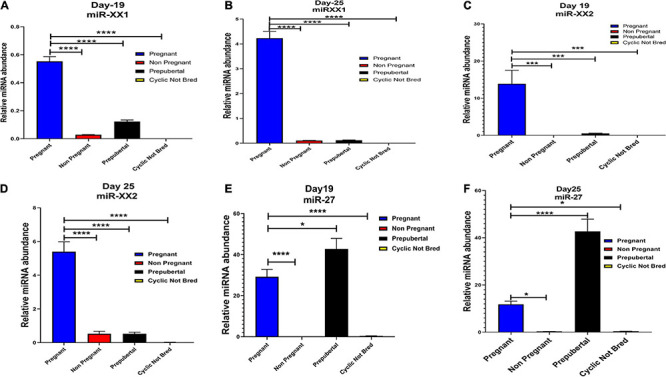
Graphical representation of the abundance of miR-XX1, miR-XX2, and miR-27 in the blood plasma of pregnant, non-pregnant, prepubertal, and cyclic heifer not bred at Day 19 and Day 25 of pregnancy. Here, X axis represents the relative miRNA abundance and Y axis represent the experimental group of animals where blue, red, black and yellow column represents pregnant, non-pregnant, prepubertal and cyclic not bred animals. *p < 0.05, ***p < 0.001, ****p < 0.0001.

Interestingly, out of 20 miRNAs, miR-195-5p, miR-708-3p, and miR-379-5p belonged to Category A (Figure 4), whereas Category B includes miR-XX1 and miR-XX2 whose high expression in the plasma of pregnant animals on both Day 19 and Day 25 was (Figures 4, 5) in accordance with the fold change analysis data of NGS. Also, their expression in the prepubertal calves and cyclic heifers was very minimal, which supports their involvement in maternal–fetal interaction via circulation (Figure 6).

Likewise, miR-130a-3p and miR-200a-3p had high expression in non-pregnant animals at Day 25 and fall under Category C (Figure 4). Nevertheless, these miRNAs are equally important, as they can be considered signatures of the implantation failure leading to the fertility issues in animals. Therefore, in such a situation, these miRNAs can be used to identify animals as non-pregnant, and further their rebreeding can be well thought in advance rather than waiting for a classical rectal palpation-based confirmation on Day 45 of pregnancy. In this way, we can trace the pregnancy in buffalo as early as Day 25 and, thus, will get an advantage of shortening the calving interval by 20 days.

High expression of category D miR-27 in pregnant and prepubertal groups (Figure 6) might be related with residual signature from the feto-maternal cross talk possibly needed for subsequent growth of the fetus. Although significant regulation could be confirmed for eight out of 20 miRNAs in the respective control groups, a few numbers of animals were tested for this observation.

### Diagnostic Value of the Top Eight Significantly Differentially Abundant MicroRNA in the Plasma of the Pregnant and Non-pregnant Buffaloes by Receiver Operating Characteristic Analysis

**FIGURE 7:**
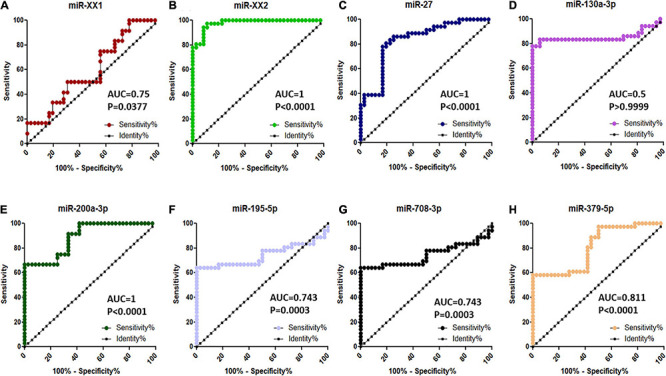
ROC curve analysis of diagnostic value of circulating miRNA for pregnant vs. non-pregnant buffaloes: **(A)** miR-XX1, **(B)** miR-XX2, **(C)** miR-27, **(D)** miR-130a-3p, **(E)** miR-200a-3p, **(F)** miR-195-5p, **(G)** miR-708-3p, and **(H)** miR-379-5p. AUC, area under the curve; ROC, receiver operating characteristic.

Among the top eight significantly differentially abundant miRNAs (miR-XX1, miR-XX2, miR-27, miR-130a-3p, miR-200a-3p, miR-195-5p, miR-708-3p, and miR-379-5p) between the pregnant and non-pregnant buffaloes, the diagnostic ability of three miRNAs (miR-XX2, miR-27, and miR-200a-3p) to differentiate pregnant and non-pregnant animals was excellent on the basis of their AUC value 1 (Figure 7). Similarly, the other four miRNAs (miR-XX1, miR-195-5p, miR-708-3p, and miR-379-5p) have good biomarker ability (AUC value > 0.7) to differentiate pregnant and non-pregnant animals. The diagnostic ability of these four miRNAs can be further improved by using them in a panel. The AUC value of miR-130a-3p was 0.5. Therefore, this miRNA may not provide clear distinction between pregnant and non-pregnant animals.

### Target Prediction of Selected MicroRNAs Using *in Silico* Approach

To identify the potential mRNA targets specific to the differentially expressed miRNA, miRanda7 tool along with target scan search engines was used. For enrichment of reproduction specific target mRNA, we used cDNA sequences of *B. bubalis* explicitly related to the ovary, fallopian tube, uterus, mammary glands, oocyte, cumulus oocyte complexes, etc. The top predicted target genes included *cyclooxygenase-2* (*COX-2*), *Talin-1* (*TLN1*), *Activin receptor type II a* (*ActRIIa*), *protein kinase AMP-activated alpha1* (*AMPKA1*) *Smad 3*, *Smad 4*, and *Smad 5*. It also included genes related to cell cycle process such as *phosphatase and tensin homolog* (*PTEN*), *cyclin B1*, and *cell division protein kinase 6* (*CDK6*). To identify the biological function of the target genes, Gene Ontology (GO) function and Kyoto Encyclopedia of Genes and Genomes (KEGG) pathway enrichment analyses have been reported. It revealed positive regulation of RNA polymerase related terms in BP followed by negative regulation of RNA polymerase, integral component of membrane, followed by “nucleus” and plasma membrane in cellular components (CCs), and finally in terms of molecular function (MF); ATP binding was followed by metal ion binding, and calcium and zinc ion 32 binding.

**FIGURE 8:**
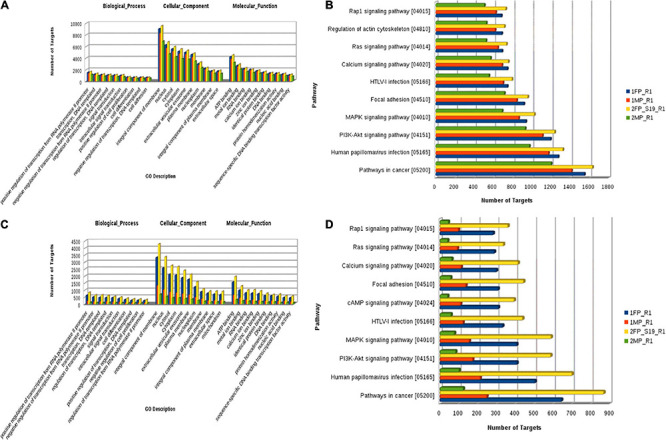
**(A)** Known microRNA: Gene Ontology categories for biological processes, cellular component, and molecular function enriched across the target genes of differentially expressed known microRNA. **(B)** Pathway distribution in known miRNA. **(C)** Novel microRNA: Gene Ontology categories for biological processes, cellular component, and molecular function enriched across the target genes of differentially expressed novel microRNA. **(D)** Pathway distribution in novel miRNA [1FP_R1, early-stage fetal placentome (cotyledon); 1MP_R1, early-stage maternal placentome (caruncle); 2FP_S19_R1, mid-stage fetal placentome (cotyledon); 2MP_R1, mid-stage maternal placentome (caruncle)].

**FIGURE 9:**
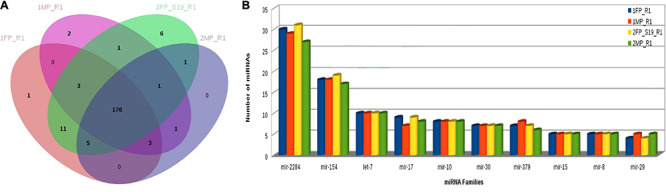
**(A)** Common miRNA families among all samples. **(B)** MiRNA family distribution across the samples.

Furthermore, using the KEGG Mapper search Pathway tool, we identified that pathways in cancer were most abundantly regulated by the target genes followed by human papillomavirus infection and PI3K-Akt signaling pathway. Almost 1,600 target genes were involved in pathways in cancer followed with 1,100 to 1,200 target genes in human papillomavirus infection and PI3K-Akt signaling pathway. Along with these, MAPK kinase signaling pathway, calcium signaling pathways, Ras signaling pathways, and pathway involved in focal adhesion were also predicted (Figure 8). Furthermore, miRNA family distribution analysis revealed that an average of 723 known miRNAs belong to almost 211 families identified. A total of 176 miRNA families (Figure 9A) were common among all the samples. Additionally, miRNA family analysis revealed that miR-2284 family was the most abundant family identified followed by miR-154 and let-7 (Figure 9B).

## Discussion

The objectives of the present work are to understand the miRNA repertoire associated with buffalo placentome during early pregnancy and mid-pregnancy and to identify the early pregnancy biomarker miRNA out of potential miRNAs from the placentome on the basis of their presence in the maternal plasma. Though various studies have proved the involvement of circulatory miRNAs in the regulation of pregnancy in cattle and buffalo (Ioannidis and Donadeu, 2016; Guelfi et al., 2017; Markkandan et al., 2018), their route of creation has not been explained at all. The present study is the first kind of study in buffaloes to identify the miRNA in maternal circulation originating from the placenta for indicating the early pregnancy.

The runs of the sequencing data from our work provided the comprehensive repertoire of the miRNA transcriptome for buffalo caruncle and cotyledon during the early pregnancy establishment phase. We used SmRNA to identify the buffalo-specific early pregnancy-associated placentome miRNA and found 2,199 miRNAs with more than 50 reads including the conserved and novel miRNAs. A similar analysis has been used in other domestic animal species like sheep (Wong et al., 2017), goat (Song et al., 2015), pig (Liu et al., 2016), horse (Pacholewska et al., 2016), camel (Jirimutu et al., 2012), and cattle (Jin et al., 2009). Though there are a total of eight published reports on buffalo miRNAs that lead to the discovery of an average of 3,013 conserved and five novel miRNAs using the deep sequencing methods (Baddela et al., 2017; Guelfi et al., 2017; Jerome et al., 2017; Guo and Guo, 2019; Lecchi et al., 2019; Singh et al., 2020), none of these studies included the miRNAs from the early pregnant placentome samples of buffaloes. The identified miRNAs in this study were further categorized into early MP (MP1), mid MP (MP2), early FP (FP1), and mid FP (FP2). Furthermore, based on differential expression analysis, we narrowed down to 20 miRNAs with significant fold change difference (log fold change 2, *p*-value and *p*-adjusted value < 0.05), identified at early stage (<25 day) and mid-stage (Days 30–35) of pregnancy in both caruncle (MP) and cotyledon (FP), which were further selected for RT-qPCR validation. The RT-qPCR validation was performed on four upregulated (miR-148a, miR-30a-5p, miR-195-5p, and miR-XX2) and four downregulated (miR-708-3p, miR-127, miR-200a-3p, and bubmiR-55) miRNAs in MP and six upregulated (miR-379-5p, miR-130a-3p, miR-1307-3p, miR-369-5p, miR-143, and miR-27) and six downregulated (miR-181b-5p, miR-23b-3p, miR-487a-3p, miR-XX1, bubmiR-1, and miR-660) miRNAs in FP. The basis for selection of both upregulated and downregulated miRNAs in feto-maternal placentomes was to provide not only complete representation to the either side of placentome but also a possible justification that the bidirectional cross talk is largely responsible for establishment of pregnancy. Additionally, it is most unlikely that all the shortlisted miRNAs can be detected in plasma either due to low copy number or may not be released in blood circulation at early stage of pregnancy. In order to catch placentome-associated miRNAs as pregnancy-specific circulatory markers, we assessed them in individual plasma samples of pregnant and non-pregnant animals. Although the qPCR data showed a minor difference in the fold change values compared with the fold change expression by NGS data, such difference could be due to the source of the biological material used for the NGS and qRT-PCR. It needs to be noted that the NGS data analysis was based on the profiling of miRNA from the placental tissue samples, whereas the RT-qPCR data were assessed on the plasma samples of animals where the copies of miRNA transcripts ultimately reached may quantitatively differ in their abundance. In addition, slight differences between the NGS and qRT-PCR data were also reported in other miRNA profiling studies of plant and animal tissues (Ioannidis and Donadeu, 2016, Ioannidis et al., 2018). Screening of these biomarkers in plasma samples has proven the robustness even over biological variability expected between panels of “screening samples” and “discovery samples.”

Among the top eight validated significantly differentially abundant miRNAs (miR-XX1, miR-XX2, miR-27, miR-130a-3p, miR-200a-3p, miR-195-5p, miR-708-3p, and miR-379-5p) between the pregnant and non-pregnant buffaloes, three miRNAs (miR-XX2, miR-27, and miR-200a-3p) were excellent (AUC value 1) biomarkers for differentiating pregnant buffaloes from non-pregnant buffaloes at the early stage of the pregnancy. In particular, significantly, an increased higher abundance of miR-XX2 in the maternal plasma of buffaloes from Day to 11 to Day 19 of the pregnancy is considered to be an excellent plasma biomarker to identify early pregnancy in buffaloes. It is intriguing to know how exactly miR-XX2 regulates the pregnancy-associated event. The miR-XX2 was reported to regulate *NOBOX* (*newborn ovary homeobox gene*), a gene critical for early embryogenesis. Although miR-XX2 was validated to target *NOBOX* for bovines (Tripurani et al., 2011), its role in early pregnancy establishment has not been explained anywhere. Therefore, recorded highest expression of miR-XX2 on Day–19 of the early pregnancy in buffaloes is the first report, and this miRNA can be used for the development of a pregnancy diagnostic assay for buffaloes. Furthermore, the plasma abundance of this miRNA in the early pregnancy of other species, like bovines, ovines, and caprines need to be studied in the future. Similarly, abundance of miR-27 in plasma can also distinguish the early pregnancy from the non-pregnancy after insemination. However, its abundance in the plasma prepubertal animals was higher than in the pregnant animals. This can be explained on the basis that miR-27 has possibly a multifunctional role as its important characteristic. Given the multifunctional trait of miRNAs, it is not surprising that miR-27 abundance was higher in prepubertal animals, and this could be largely responsible for its interaction with the multiple target genes. Nevertheless, this scenario would not undermine the diagnostic ability of miR-27 as an early pregnancy biomarker, as the diagnosis of early pregnancy is important in post-pubertal animals after insemination. Supporting our observation on buffaloes, miR-27 plasma abundance was also observed in the early pregnancy of cattle (Ioannidis and Donadeu, 2016), highlighting its role in trophoblast differentiation and vascularization during placenta formation (Su et al., 2010).

Interestingly, the higher abundance of miR-200a-3p in the plasma of non-pregnant animals compared with the pregnant animals on Days 19 and 25 would help in selecting non-pregnant animals, so that farmers can monitor the buffaloes for estrus symptoms for insemination. Hence, this study also provides a biomarker for non-pregnant animal identification. It has been found that miR-200a-3p regulates the *PTEN*-mediated proliferation and apoptosis of the cells (Reza et al., 2019); and thus, its high expression might be responsible for PTEN-mediated inhibition of epithelial cell proliferation, thus effecting implantation. Furthermore, miR-200c, an isomiR of miR-200a-3p, was found to regulate gene *Talin*, a key component of focal adhesion and important regulator of integrin activation (Calderwood et al., 1999). Furthermore, it can be explained that higher expression of miR-200 family in the non-pregnant group in our study may in turn regulate both the *Talin* and *integrin* activation, inhibiting the cell adhesion process and consequently promoting the chances of implantation failure in buffaloes. However, future functional studies are needed in support of this aspect.

In addition to the above-mentioned three promising miRNAs (miR-XX2, miR-27, and miR-200a-3p), the plasma abundance of the other four miRNAs (miR-XX1, miR-195-5p, miR-708-3p, and miR-379-5p) has good biomarker ability (AUC value > 0.7) to be a part of the biomarker panel to differentiate pregnant and non-pregnant buffaloes. With the use of target prediction, it was found that miR-XX1 regulates *COX-2*, a gene critical for implantation in mice (Chakrabarty et al., 2007). In ruminants, *COX-2* regulates the *PGFS*-mediated synthesis of *PGF2*α to promote luteolysis (Arosh et al., 2002). The increased expression of miR-XX1 can be explained that regulation of *COX-2* through miR-XX1 might inhibit the production of *PGF2*α and thus prevents luteolysis from happening. Therefore, the higher expression of miR-XX1 at Day 25 pregnant plasma samples is plausibly related with its regulatory response toward *COX-2* gene for promoting pregnancy establishment in buffalo. The miR-195-5p, miR-708-3p, and miR-379-5p were also identified as drastically upregulated at Day 19 pregnant plasma during early pregnancy than in the non-pregnant group. MiR-195-5p might be associated with the regulation of trophoblast fusion to the endometrium via *ActIIRA*, a type II receptor for several members of the *TGF-*β superfamily, including Nodal. Also, target prediction predicted several extracellular matrix metalloproteinases (*MMP*s) as the potent target mRNA against miR-195-5p, which can be exploited further to delineate its role in trophoblast cell fusion in buffaloes (Aoki-Kinoshita and Kanehisa, 2007). To date, no functional validation studies are performed in bovines for miR-708-3p and miR-379-5p. Consequently, their role in buffalo early pregnancy establishment needs further investigations.

Notably, the expression of miR-181b-5p, miR-148a, miR-23b-3p, miR-379-5p, and miR-369-5p was found higher in the pregnant group as compared with the non-pregnant group, but their presence in the plasma of prepubertal calves and cyclic heifers restricted them to be considered as pregnancy-specific biomarkers. It was reported that miR-181-associated target genes, such as *SSP1*, *ITGB-3*, and *ESR1*, were known to be involved in remodeling of the endometrium in pigs (White et al., 2005; Erikson et al., 2009). A study implicated by Ioannidis et al. (2018) found that miR-148 might be involved in bovine estrous cycle. Another study reported that both miR-148a and miR-30a are mammary gland-associated miRNAs, and their high expression in milk can be used for pregnancy detection in bovines (Schanzenbach et al., 2017). The expression level of cell free-mature miR-148a and miR-15b was associated with metritis in cattle (Kasimanickam and Kastelic, 2016). Additionally, miR-30c was reported to target *CDK-12* and downregulate *DDR* genes, thus inducing the cellular apoptosis in blastocyst and therefore hampered the preimplantation in bovine (Lin et al., 2019). Both miR-30a and miR-30c belong to the miR-30 family. The higher plasma levels of miR-30a in the non-pregnant group at Day 19 in the present study need to be explored further to see if it is functioning in the same way in buffaloes via *DDR* genes, thus causing the pregnancy failure due to hampered preimplantation. Similarly, miR-127, an endometrial miRNA, was reported to play an important role during mesodermal lineage of embryonic stem cells by targeting *left–right determination factor 2* (*Lefty2*) and regulating *Nodal* signaling (Ma et al., 2016). Also, its expression was found in eight-cell staged embryos in bovine. However, its higher expression was observed in non-pregnant animal plasma at Day 25 as compared with the pregnant animals in the present study.

Taking together all observations in the present study, seven miRNAs, i.e., miR-XX1, miR-XX2, miR-27, miR-200a-3p, miR-195-5p, miR-708-3p, and miR-379-5p, can be used as a biomarker panel to distinguish pregnant and non-pregnant animals in less than 25 days of the pregnancy. Nevertheless, as one miRNA can control so many genes and vice versa, there is a need of exhaustive functional validation studies for establishing a conclusive involvement of these miRNAs within buffalo pregnancy.

## Conclusion

In conclusion, the present study profiled the entire miRNA repertoire of early and mid FP and MP in buffaloes and established the presence of placental miRNA in the maternal plasma. In addition, the study provided seven miRNAs (miR-XX1, miR-XX2, miR-27, miR-200a-3p, miR-195-5p, miR-708-3p, and miR-379-5p) as potential biomarkers for differentiating pregnant and non-pregnant buffaloes on or before the 25th day of pregnancy. Particularly, the abundance of miR-XX2 in the maternal plasma of buffaloes from Day 11 to Day 19 of the pregnancy was established an excellent plasma biomarker to identify early pregnancy in buffaloes. Simultaneously, confirmation of higher abundance of miR-200a-3p in the plasma of non-pregnant animals compared with the pregnant animals on the Days 19 and 25 would help in screening of non-pregnant animals for re-breeding. As a follow-up of this study, the promising miRNA candidates can be suitably taken up for the development of early pregnancy diagnosis kits after validating the plasma abundance of these miRNAs in a large population of buffaloes. In addition, future functional validation studies are required to understand the comprehensive pregnancy-associated biological functions of the miRNAs as well as to establish the pregnancy diagnosis kits in the market in an authentic manner.

## Data Availability Statement

All data generated or analysed during this study are included in this published article and its supplementary information files. The miRNA sequence data has been submitted to NCBI repository with accession to cite for these SRA data as: PRJNA705293 and having biosample details as follows: BioSample: SAMN18080043, SAMN18080044, SAMN18080045, and SAMN18080046.

## Ethics Statement

The animal study was reviewed and approved by Institutional Animal Ethics Commitee, ICAR-National Dairy Research Institute, Karnal, India.

## Author Contributions

TD conceived, designed, and supervised the work. PS performed the experiments, interpreted the results, and wrote the first draft of the manuscript. MR, KS, MI, SJ, and RC performed bioinformatics analysis and interpretation. DK and TD did the biological data interpretation. AS, PD, and AP helped in sample preparation and experiments. RK, AK, and SO provided vital inputs to this study. MI, SJ, DK, RK, and TD edited the final manuscript. All authors contributed to the article and approved the submitted version.

## Conflict of Interest

The authors declare that the research was conducted in the absence of any commercial or financial relationships that could be construed as a potential conflict of interest.

## Publisher’s Note

All claims expressed in this article are solely those of the authors and do not necessarily represent those of their affiliated organizations, or those of the publisher, the editors and the reviewers. Any product that may be evaluated in this article, or claim that may be made by its manufacturer, is not guaranteed or endorsed by the publisher.

## Footnotes

1
https://www.bioinformatics.babraham.ac.uk/projects/fastqc/

2
http://hannonlab.cshl.edu/fastx_toolkit/

3
https://www.ncbi.nlm.nih.gov/assembly/GCF_003121395.1

4
http://www.mirbase.org/ftp.shtml

5
www.miRBase.org

## Supplementary Material

Supplementary Figure 1 Venn diagram for miRNAs identified in early and mid stage sample of maternal placentome.

Supplementary Figure 2 Venn diagram for miRNAs identified in early and mid stage sample of fetal placentome.

Supplementary Figure 3 Heat map for the differential abundance of miRNA between the early and mid stage fetal placentome.

Supplementary Figure 4 Heat map for the differential abundance of miRNA between the early and mid stage maternal placentome.

Supplementary Figure 5 Examples of secondary structure prediction of Novel microRNAs in fetal placentome.

Supplementary Figure 6 Examples of secondary structure prediction of Novel microRNAs in maternal.

Supplementary Figure 7 Relative miRNA abundance of miR-379-5p, miR-195-5p, and miR-708-3p in the blood plasma of pregnant, non-pregnant, prepubertal, and cyclic heifers not bred at day-19 and day-25 of pregnancy.

Supplementary Figure 8 Relative miRNA abundance of miR-130a-3p and miR-200a-3p in the blood plasma of pregnant, non-pregnant, prepubertal, and cyclic heifers not bred at day-19 and day-25 of pregnancy.

Supplementary Table 1 The summary of raw and trimmed reads used for the analysis.

Supplementary Table 2 Total number of Identified miRNA.

Supplementary Table 3 List of Primers.

Supplementary Table 4 List of top 20 differentially expressed miRNAs with their associated placentome origin.

Supplementary Table 5 Details of top 20 differentially expressed miRNA with minimum fold change of 2 (*p* ≤ 0.05) and (*p*-adj ≤ 0.05).
