# Supplementary figures and images for: Establishment of Repertoire of Placentome-Associated MicroRNAs and Their Appearance in Blood Plasma Could Identify Early Establishment of Pregnancy in Buffalo (Bubalus bubalis)
Source: Front Cell Dev Biol. 2021 Aug 26;9:673765. doi: 10.3389/fcell.2021.673765 (PMC8427669; doi:10.3389/fcell.2021.673765)

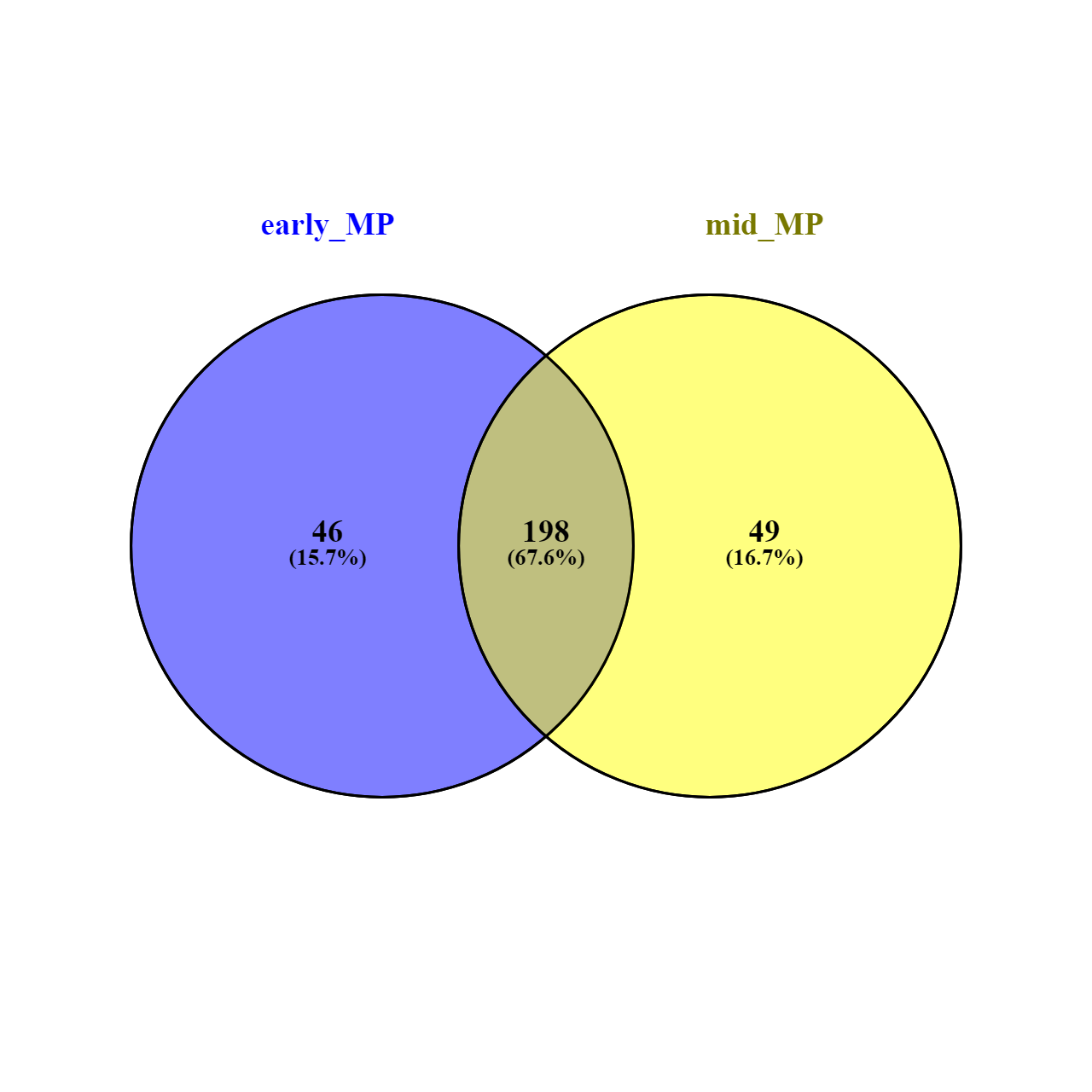

Supplement: Supplementary Figure 1 — Venn diagram for miRNAs identified in early and mid stage sample of maternal placentome. [file Image_1.TIF]

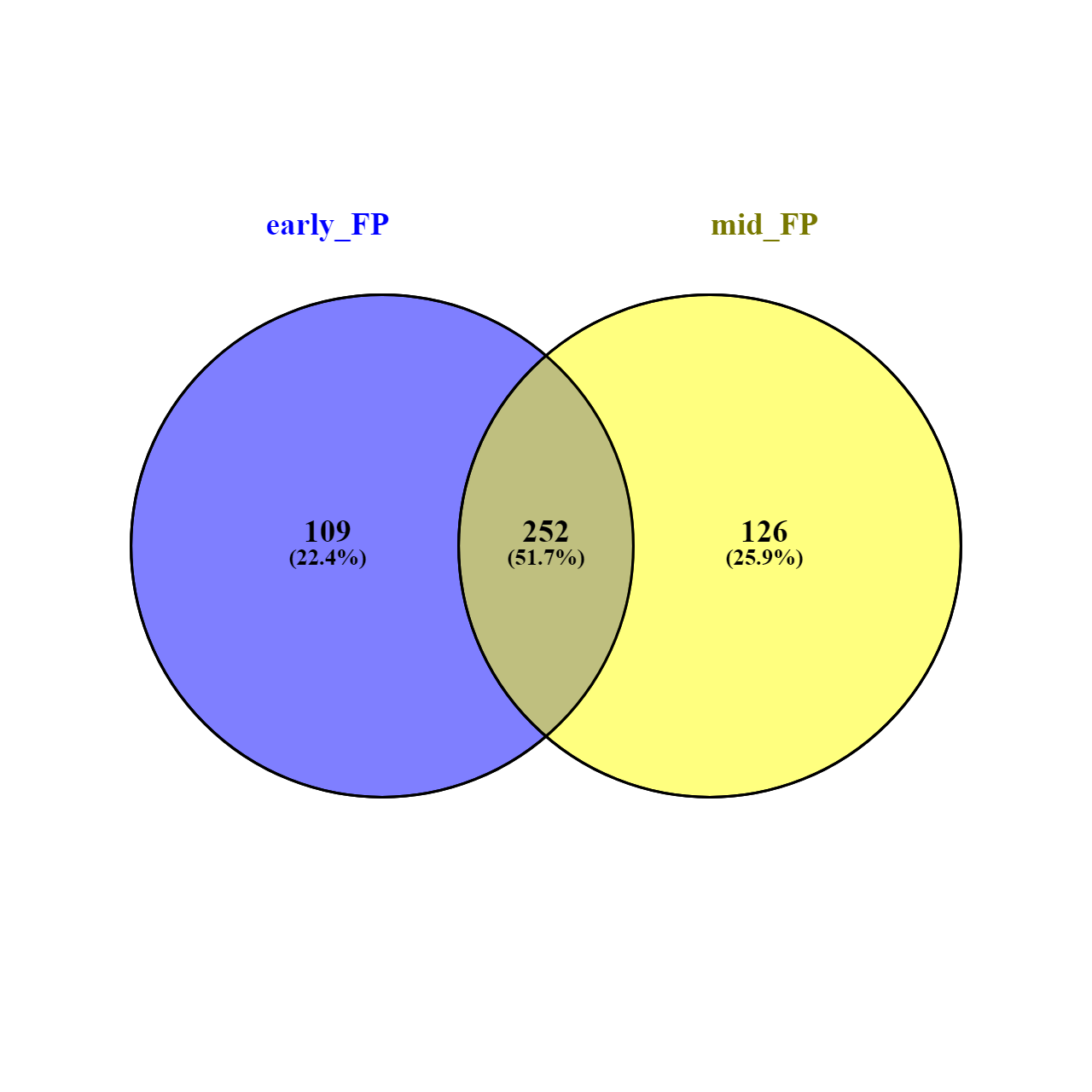

Supplement: Supplementary Figure 2 — Venn diagram for miRNAs identified in early and mid stage sample of fetal placentome. [file Image_2.TIF]

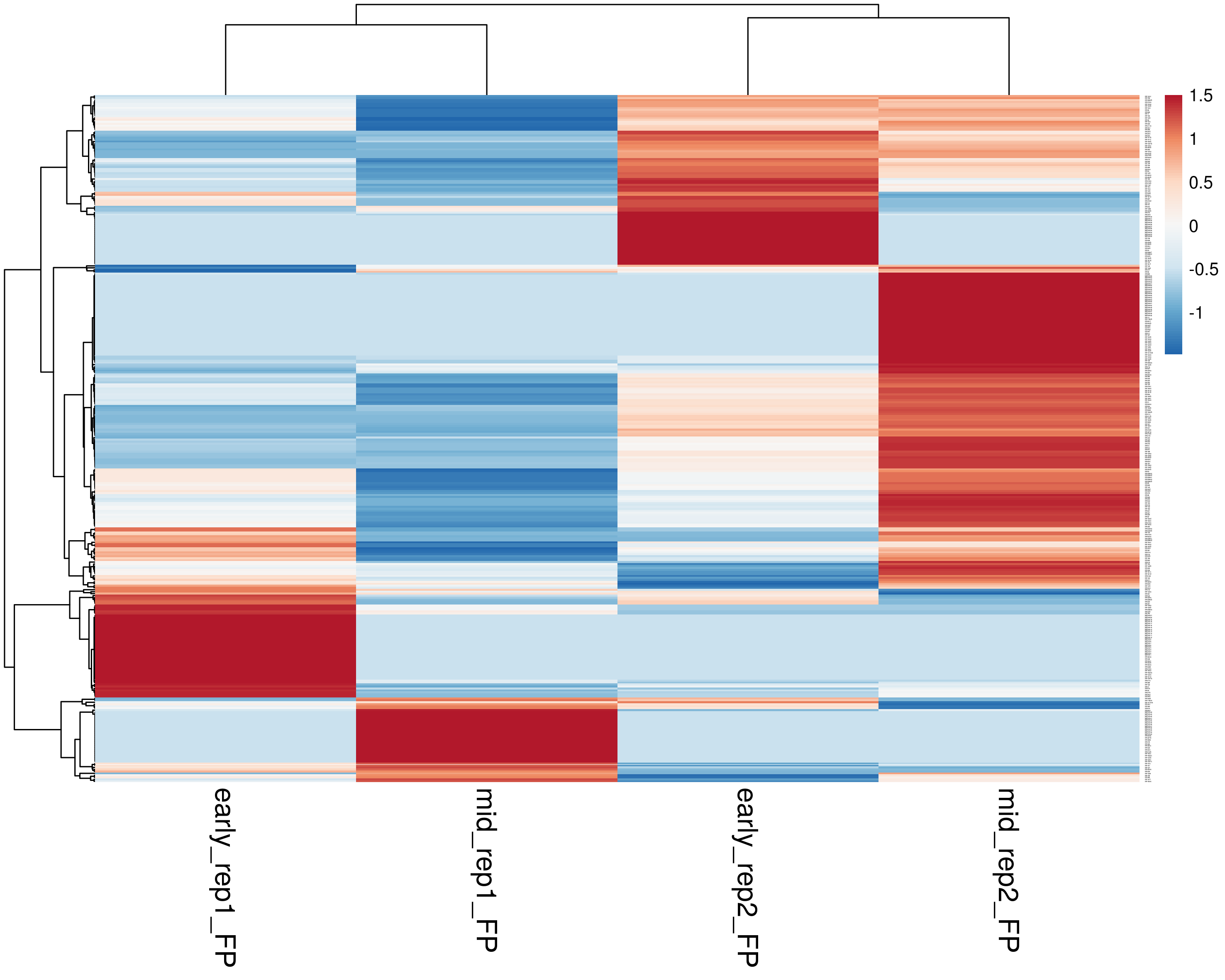

Supplement: Supplementary Figure 3 — Heat map for the differential abundance of miRNA between the early and mid stage fetal placentome. [file Image_3.TIF]

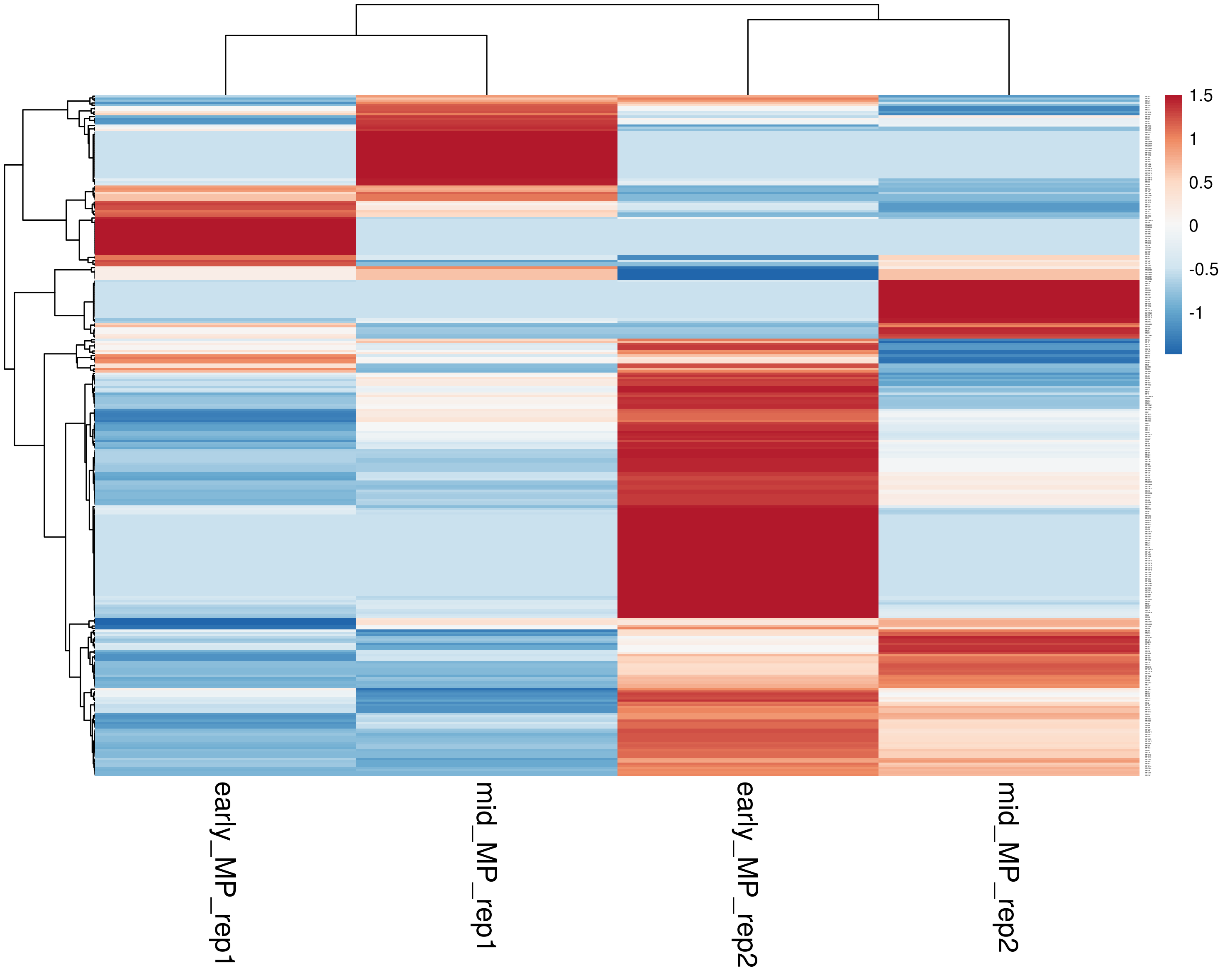

Supplement: Supplementary Figure 4 — Heat map for the differential abundance of miRNA between the early and mid stage maternal placentome. [file Image_4.TIF]

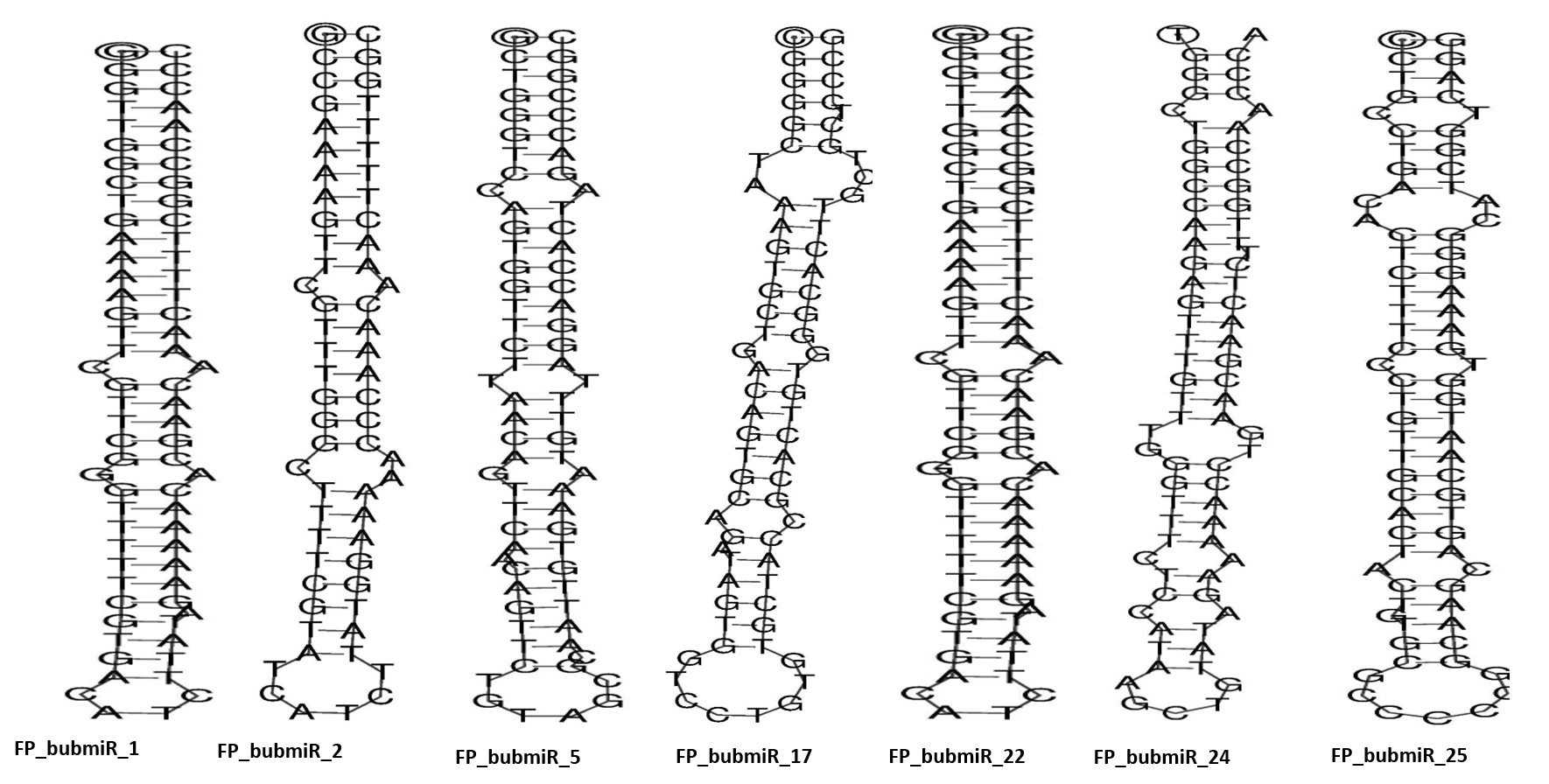

Supplement: Supplementary Figure 5 — Examples of secondary structure prediction of Novel microRNAs in fetal placentome. [file Image_5.TIF]

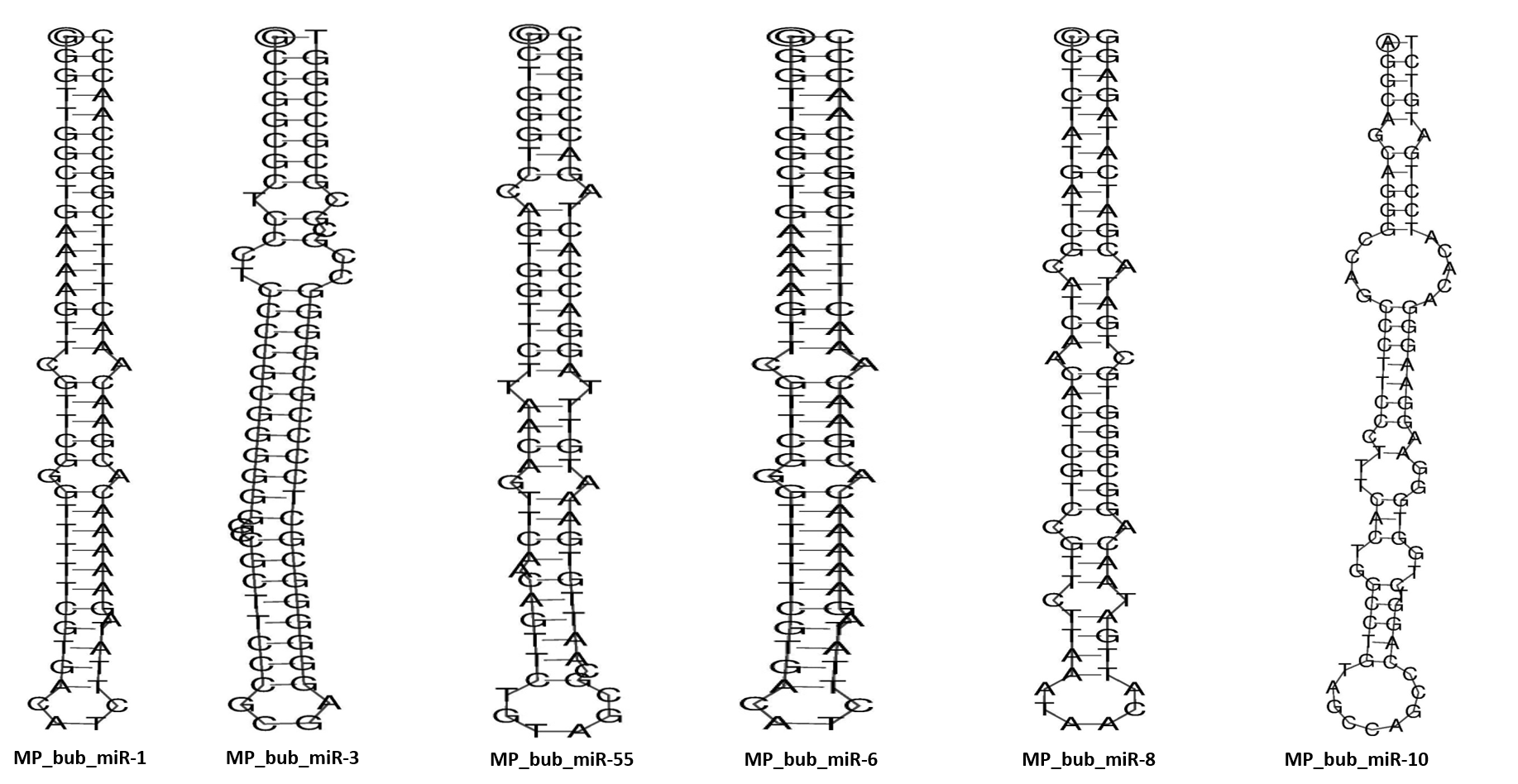

Supplement: Supplementary Figure 6 — Examples of secondary structure prediction of Novel microRNAs in maternal. [file Image_6.TIF]

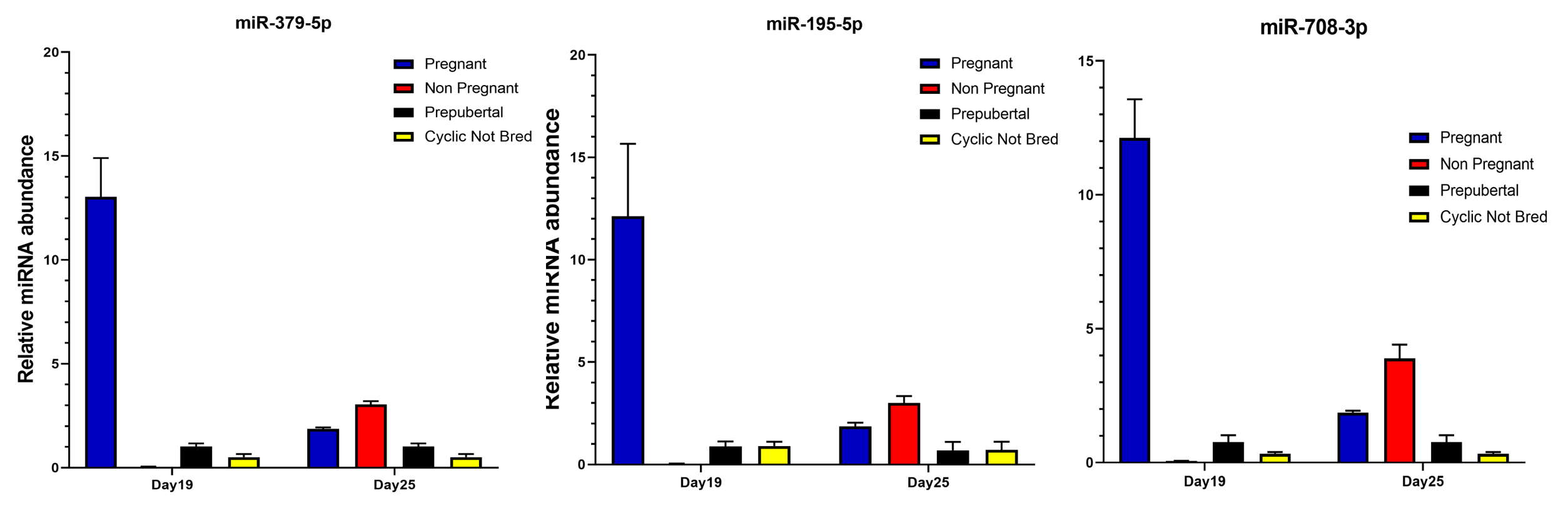

Supplement: Supplementary Figure 7 — Relative miRNA abundance of miR-379-5p, miR-195-5p, and miR-708-3p in the blood plasma of pregnant, non-pregnant, prepubertal, and cyclic heifers not bred at day-19 and day-25 of pregnancy. [file Image_7.TIF]

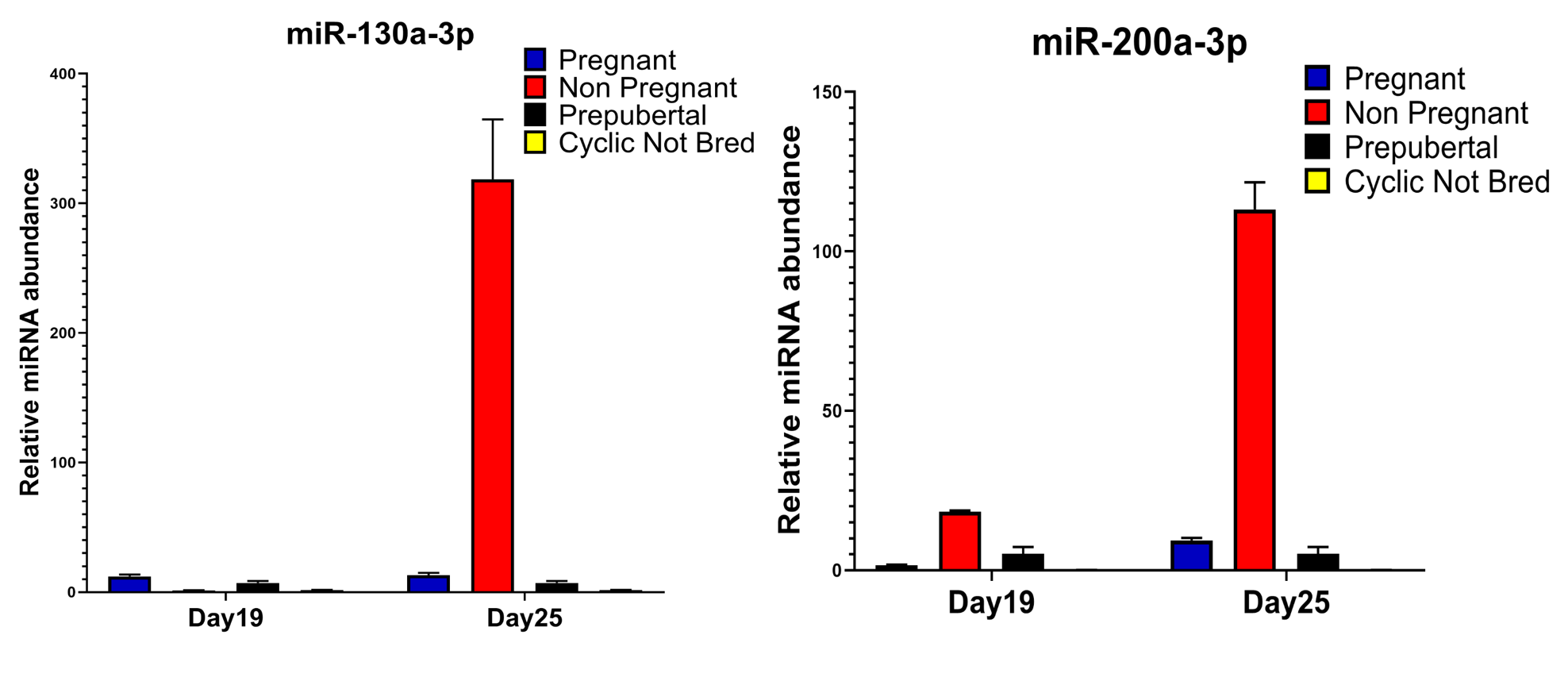

Supplement: Supplementary Figure 8 — Relative miRNA abundance of miR-130a-3p and miR-200a-3p in the blood plasma of pregnant, non-pregnant, prepubertal, and cyclic heifers not bred at day-19 and day-25 of pregnancy. [file Image_8.TIF]
