# Supplementary material for: Establishment of Repertoire of Placentome-Associated MicroRNAs and Their Appearance in Blood Plasma Could Identify Early Establishment of Pregnancy in Buffalo (Bubalus bubalis)
Source: Front Cell Dev Biol. 2021 Aug 26;9:673765. doi: 10.3389/fcell.2021.673765 (PMC8427669; doi:10.3389/fcell.2021.673765)
Supplement: Supplementary Table 1 — The summary of raw and trimmed reads used for the analysis. [file Table_1.DOCX]

**Supplementary table 1. The summary of raw and trimmed reads used for the analysis**

| **Sample Name** | **Raw Reads** | **Filter Reads** | **Read Aligned** |
| --- | --- | --- | --- |
| **Early_FP_rep1** | **19448614** | **6077215** | **5558302** |
| **Early_FP_rep2** | **18000340** | **6526150** | **5954070** |
| **Mid_FP_rep1** | **16866486** | **5206394** | **4793433** |
| **Mid_FP_rep2** | **19168506** | **5655549** | **5014304** |
| **Early_MP_rep1** | **19235915** | **13137731** | **12820726** |
| **Early_MP_rep2** | **16465915** | **9659928** | **9135566** |
| **Mid_MP_rep1** | **16316004** | **11316881** | **11052389** |
| **Mid_MP_rep2** | **12964241** | **6599074** | **6198384** |

(***Early_FP corresponds to early fetal cotyledon (fetal Placentome), Mid_FP corresponds to Mid fetal Cotyledon (fetal Placentome), Early_MP corresponds to early maternal caruncle (maternal placentome), Mid_FP corresponds to mid maternal caruncle (maternal placentome), rep 1 and 2 corresponds to replicate 1 and replicate 2)**
