# Supplementary material for: Establishment of Repertoire of Placentome-Associated MicroRNAs and Their Appearance in Blood Plasma Could Identify Early Establishment of Pregnancy in Buffalo (Bubalus bubalis)
Source: Front Cell Dev Biol. 2021 Aug 26;9:673765. doi: 10.3389/fcell.2021.673765 (PMC8427669; doi:10.3389/fcell.2021.673765)
Supplement: Supplementary Table 2 — Total number of Identified miRNA. [file Table_2.DOCX]

**Supplementary table 2 Identified miRNA**

| **Sample Name** | **Total miRNA** | **Known miRNA** | **Novel miRNA** | **Novel miRNA with star(*)** |
| --- | --- | --- | --- | --- |
| **Early_FP_rep1** | **340** | **204** | **136** | **21** |
| **Early_FP_rep2** | **297** | **217** | **80** | **11** |
| **Mid_FP_rep1** | **303** | **184** | **119** | **12** |
| **Mid_FP_rep2** | **323** | **215** | **108** | **21** |
| **Early_MP_rep1** | **226** | **187** | **39** | **5** |
| **Early_MP_rep2** | **240** | **219** | **21** | **5** |
| **Mid_MP_rep1** | **225** | **190** | **35** | **5** |
| **Mid_MP_rep2** | **245** | **204** | **41** | **6** |
| **Total** | **2199** | **1620** | **579** | **86** |
