# Supplementary material for: Establishment of Repertoire of Placentome-Associated MicroRNAs and Their Appearance in Blood Plasma Could Identify Early Establishment of Pregnancy in Buffalo (Bubalus bubalis)
Source: Front Cell Dev Biol. 2021 Aug 26;9:673765. doi: 10.3389/fcell.2021.673765 (PMC8427669; doi:10.3389/fcell.2021.673765)
Supplement: Supplementary Table 3 — List of Primers. [file Table_3.DOCX]

**Supplementary Table 4 List of Primers**

| **Chromosomal location** | **miRNA** | **Mature Sequence** | **Orientation** | **Primer Sequence** |
| --- | --- | --- | --- | --- |
| **Chrm 3** | **miR-XX1** | **GTACAGTACTGTGATAACTGAA** | **- [Negative strand]** | **AAGGTACAGTACTGTGATAACTGA A** |
| **Chrm16** | **miR-130a-3p** | **CAGTGCAATGTTAAAAGGGCA** | **- [Negative strand]** | **GCA GTG CAA TGT TAA AAG GGC ATT GG** |
| **Chrm23** | **miR-1307-3p** | **CTCGGCGTGGCGTCGGTCGTGG** | **- [Negative strand]** | **GTG GCG TCG GTC GTG GTA G** |
| **Chrm5** | **miR-181b-5p** | **AACATTCATTGCTGTCGGTGGG** | **+ [Positive strand]** | **AACATTCATTGCTGTCGGTGGG** |
| **Chrm3** | **miR-23b-3p** | **ATCACATTGCCAGGGATTACCAC** | **+ [Positive strand]** | **ATCACATTGCCAGGGATTACCAC** |
| **Chrm20** | **miR-369-5p** | **AGATCGACCGTGTTATATTCG** | **- [Negative strand]** | **AGATCGACCGTGTTATATTCG** |
| **Chrm20** | **miR-379-5p** | **TGGTAGACTATGGAACGTAGG** | **- [Negative strand]** | **GAG ATG GTA GAC TAT GGA ACG TAG G** |
| **Chrm20** | **miR-487a-3p** | **AATCATACAGGGACATCCAGTT** | **- [Negative strand]** | **GAC GAA TCA TAC AGG GAC ATC CAG TT** |
| **Chrm11** | **bub-miR-1** | **TGAAAAGTTCGTTCGGGTTTTT** | **+ [Positive strand]** | **TGAAAAGTTCGTTCGGGTTTTT** |
| **Chrm20** | **bub-miR-55** | **TGAAATGTTTAGGACCACTAGA** | **- [Negative strand]** | **TGAAATGTTTAGGACCACTAGA** |

| **Chromosome** | | **miRNA** | **Mature Sequence** | | **Orientation** | **Primer Sequence** | |
| --- | --- | --- | --- | --- | --- | --- | --- |
| **Chrm 8** | **miR-148a** | | | **TCAGTGCACTACAGAACTTTGTC** | **+ [Positive strand]** | **AAG TCA GTG CAC TAC AGA ACT TTG TC** |  |
| **Chrm 10** | **miR-30a-5p** | | | **TGTAAACATCCTCGACTGGAAGC** | **+ [Positive strand]** | **TGTAAACATCCTCGACTGGAAGC** |  |
| **Chrm 3** | **miR-195-5p** | | | **TAGCAGCACAGAAATATTGGC** | **+ [Positive strand]** | **TAG CAG CAC AGA AAT ATT GGC ACT** |  |
| **Chrm 8** | **miR-XX2** | | | **TAGGTAGTTTCCTGTTGTTGGG** | **+ [Positive strand]** | **TAGGTAGTTTCCTGTTGTTGGG** |  |
| **Chrm 5** | **miR-708-3p** | | | **CATCTAGACTGTGAGCTTCTAGA** | **+ [Positive strand]** | **GCAT CTA GAC TGT GAG CTT CTA GA** |  |
| **Chrm 5** | **miR-200a-3p** | | | **TAACACTGTCTGGTAACGATGTT** | **+ [Positive strand]** | **TAACACTGTCTGGTAACGATG** |  |
| **Chrm 3** | **miR-27** | | | **TTCACAGTGGCTAAGTTCTGCACCT** | **+ [Positive strand]** | **TTCACAGTGGCTAAGTTCTGC** |  |
| **Chrm 20** | **miR-127** | | | **TCGGATCCGTCTGAGCTTGGCT** | **- [Negative strand]** | **TCGGATCCGTCTGAGCTTGGCT** |  |
| **Chrm X** | **miR-660** | | | **TACCCATTGCATATCGGAGCTG** | **- [Negative strand]** | **TACCCATTGCATATCGGAGCTG** |  |
| **Chrm 9** | **miR-143** | | | **TGAGATGAAGCACTGTAGCTC** | **- [Negative strand]** | **TGAGATGAAGCACTGTAGCTC** |  |
| **Endogeneous Control miRNA** | **btamiR-103** | | | **NA** |  | **GCAGAGCAGCATTGTACAG** |  |
| **Endogeneous Control miRNA** | **btamiR-423-5p** | | | **NA** |  | **CAGTGAGGGGCAGAGAG** |  |
