# Supplementary material for: Establishment of Repertoire of Placentome-Associated MicroRNAs and Their Appearance in Blood Plasma Could Identify Early Establishment of Pregnancy in Buffalo (Bubalus bubalis)
Source: Front Cell Dev Biol. 2021 Aug 26;9:673765. doi: 10.3389/fcell.2021.673765 (PMC8427669; doi:10.3389/fcell.2021.673765)
Supplement: Supplementary Table 4 — List of top 20 differentially expressed miRNAs with their associated placentome origin. [file Table_4.DOCX]

**Supplementary table 3 List of top 20 differentially expressed miRNAs with their associated placentome origin**

| **miRNA** | **Origin** |
| --- | --- |
| miR-379-5p | Fetal Placentome |
| miR-130a-3p | Fetal Placentome |
| miR-1307-3p | Fetal Placentome |
| miR-369-5p | Fetal Placentome |
| miR-181b-5p | Fetal Placentome |
| miR-23b-3p | Fetal Placentome |
| miR-487a-3p | Fetal Placentome |
| miR-XX1 | Fetal Placentome |
| bubmiR-1 | Fetal Placentome |
| miR-143 | Fetal Placentome |
| miR-660 | Fetal Placentome |
| miR-27 | Fetal Placentome |
| miR-148a | Fetal Placentome |
| miR-30a-5p | Maternal Placentome |
| miR-195-5p | Maternal Placentome |
| miR-708-3p | Maternal Placentome |
| miR-127 | Maternal Placentome |
| miR-200a-3p | Maternal Placentome |
| bubmiR-55 | Maternal Placentome |
| miR-XX2 | Maternal Placentome |
