# Supplementary material for: Establishment of Repertoire of Placentome-Associated MicroRNAs and Their Appearance in Blood Plasma Could Identify Early Establishment of Pregnancy in Buffalo (Bubalus bubalis)
Source: Front Cell Dev Biol. 2021 Aug 26;9:673765. doi: 10.3389/fcell.2021.673765 (PMC8427669; doi:10.3389/fcell.2021.673765)
Supplement: Supplementary Table 5 — Details of top 20 differentially expressed miRNA with minimum fold change of 2 (p ≤ 0.05) and (p-adj ≤ 0.05). [file Table_5.DOCX]

| **Chromosome**  **Supplementary Table** **5.** Details of top 20 differentially expressed miRNA with minimum fold change of 2 (p ≤0.05) and (p-adj≤0.05) | **miRNA** | **Hairpin sequence** | **Mature Sequence** | **Log2Fold change** | **p-value** | **P-adj BY** | **P-adj BH** | **P-adj Holm** | **Orientation** |
| --- | --- | --- | --- | --- | --- | --- | --- | --- | --- |
| Chrm 8 | miR-148a | TCTTTTGAGGCAAAGTTCTGAGACACTCCGACTCTGAATATGATAGAAGTCAGTGCACTACAGAACTTTGTCTCTAGGGA | TCAGTGCACTACAGAACTTTGTC | 28.83718 | 1.67E-09 | 1.16E-12 | 4.18E-12 | 8.35E-11 | + [Positive strand] |
| Chrm5 | miR-181b-5p | CATTCATTGCTGTCGGTGGGTTGAACTGTGTGGACAAGCTCACTGAACAATGAATG | AACATTCATTGCTGTCGGTGGG | -25.61 | 9.41E-09 | 0.00E+00 | 2.35E-11 | 4.95E-10 | + [Positive strand] |
| Chrm11 | bub-miR-1 | GGGTTGGCTGAAAAGTTCGTTCGGGTTTTTCGTGACATCTTATAGAAAAACACGAACAAACTTTTCGGCCAACCC | TGAAAAGTTCGTTCGGGTTTTT | -24.434 | 2.62E-08 | 0.00E+00 | 6.55E-11 | 1.46E-09 | + [Positive strand] |
| Chrm3 | miR-23b-3p | TGGCTGCTTGGGTTCCTGGCATGCTGATTTGTGACTTAAGATTAAAATCACATTGCCAGGGATTACCACGCAACCA | ATCACATTGCCAGGGATTACCAC | -23.809 | 4.45E-08 | 0.00E+00 | 1.11E-10 | 2.62E-09 | + [Positive strand] |
| Chrm 10 | miR-30a-5p | GCGGCTGTAAACATCCTCGACTGGAAGCTGTGAGGCTGCAGAAAGGCTTTCAGTCGGATGTTTGCAGCTGC | TGTAAACATCCTCGACTGGAAGC | 26.12156 | 4.78E-08 | 0.00E+00 | 1.20E-10 | 2.99E-09 | + [Positive strand] |
| Chrm20 | miR-379-5p | GAGATGGTAGACTATGGAACGTAGGCTTTGTGATTTTTGACCTATGTAACATGGTCCACTAACTC | TGGTAGACTATGGAACGTAGG | 23.6918 | 8.46E-08 | 0.00E+00 | 2.12E-10 | 5.64E-09 | - [Negative strand] |
| Chrm20 | bub-miR-55 | GCTGGGTCCAGTGGTTCTTAACAGTTCAACAGTTCTGTAGCGCAATTGTGAAATGTTTAGGACCACTAGACCCGGC | TGAAATGTTTAGGACCACTAGA | 22.6289 | 8.83E-08 | 0.00E+00 | 2.21E-10 | 6.31E-09 | - [Negative strand] |
| Chrm 8 | miR-XX2 | GGTGATTTAGGTAGTTTCCTGTTGTTGGGATCCACCTTTCTCTCGACAGCACGACACTGCCTTCATTACT | TAGGTAGTTTCCTGTTGTTGGG | 25.0845 | 1.58E-07 | 0.00E+00 | 3.95E-10 | 1.22E-08 | + [Positive strand] |
| Chrm 5 | miR-708-3p | CTGCCCTCAAGGAGCTTACAATCTAGCTGGGGGTAAACGACTTGCACATGAACGCATCTAGACTGTGAGCTTCTAGAGGGCAG | CATCTAGACTGTGAGCTTCTAGA | -24.9329 | 1.88E-07 | 0.00E+00 | 4.70E-10 | 1.57E-08 | + [Positive strand] |
| Chrm 3 | miR-195-5p | AGCAGCACAGAAATATTGGCACTGGGAAGAAAGCCTGCCAATATTGGCTGTGCTGCT | TAGCAGCACAGAAATATTGGC | 24.61854 | 2.67E-07 | 0.00E+00 | 6.68E-10 | 2.43E-08 | + [Positive strand] |
| Chrm 5 | miR-200a-3p | GGACATCTTACCGGACAGTGCTGGATTTCTCGGCTCGACTCTAACACTGTCTGGTAACGATGTTC | TAACACTGTCTGGTAACGATGTT | -8.89036 | 2.53E-05 | 0.00E+00 | 6.33E-08 | 2.53E-06 | + [Positive strand] |
| Chrm20 | miR-487a-3p | TGAAGAGTGGTTATCCCTGCTGTGTTCGCTGTATTTATGACGAATCATACAGGGACATCCAGTTTTTCA | AATCATACAGGGACATCCAGTT | -8.21147 | 0.00414 | 0.00E+00 | 1.04E-05 | 4.60E-04 | - [Negative strand] |
| Chrm X | miR-660 | CTCCCGTACCCATTGCATATCGGAGCTGTGAATTCTCAAAGCACCTCCTATGTGCATGGATTACAGGAG | TACCCATTGCATATCGGAGCTG | -0.11309 | 0.009 | 0.00E+00 | 2.25E-05 | 1.13E-03 | - [Negative strand] |
| Chrm 9 | miR-143 | CCCAGCCTGAGGTGCAGTGCTGCATCTCTGGTCAGTTGGGAGTCTGAGATGAAGCACTGTAGCTCGGGAAGGG | TGAGATGAAGCACTGTAGCTC | 0.043171 | 0.009 | 0.00E+00 | 2.25E-05 | 1.29E-03 | - [Negative strand] |
| Chrm 3 | miR-27 | AGGTGCAGAGCTTAGCTGATTGGTGAACAGTGACTGGTTTCCGCTTTGTTCACAGTGGCTAAGTTCTGCACCT | TTCACAGTGGCTAAGTTCTGC | 2.4296 | 0.0099 | 0.00E+00 | 2.48E-05 | 1.65E-03 | + [Positive strand] |
| Chrm 20 | miR-127 | TCCAGCCTGCTGAAGCTCAGAGGGCTCTGATTCAGAAAGATCATCGGATCCGTCTGAGCTTGGCTGGTCGGA | TCGGATCCGTCTGAGCTTGGCT | -0.47645 | 0.0099 | 0.00E+00 | 2.48E-05 | 1.98E-03 | - [Negative strand] |
| Chrm16 | miR-130a-3p | GCGGGCCGGGGCTCTTTTCACATTGTGCTACTGTCTGCGCCTGTCACTAGCAGTGCAATGTTAAAAGGGCATTGGCCGC | CAGTGCAATGTTAAAAGGGCA | 9.366308 | 3.17E-02 | 0.00E+00 | 7.92E-05 | 7.92E-03 | - [Negative strand] |
| Chrm23 | miR-1307-3p | CTGCCTACCAATCTCGACCGGACCTCGACCGGCTCGTCTATATTGCCAATCGACTCGGCGTGGCGTCGGTCGTGGTAGATAG | CTCGGCGTGGCGTCGGTCGTGG | 9.255185 | 0.033715 | 0.00E+00 | 8.43E-05 | 1.12E-02 | - [Negative strand] |
| Chrm 3 | miR-XX1 | CCTTTTTCGGTTATCATGGTACCGATGCTGTATATCTGAAAGGTACAGTACTGTGATAACTGAAGAATGG | GTACAGTACTGTGATAACTGAA | -9.02371 | 0.036682 | 0.00E+00 | 9.17E-05 | 1.83E-02 | - [Negative strand] |
| Chrm20 | miR-369-5p | CTGAAGGGAGATCGACCGTGTTATATTCGCTTTATTGACTTCGAATAATACATGGTTGATCTTTTCTCAG | AGATCGACCGTGTTATATTCG | 8.740084 | 0.045026 | 0.00E+00 | 1.13E-04 | 4.50E-02 | - [Negative strand] |
